# Exploiting metabolic adaptations to overcome dabrafenib treatment resistance in melanoma cells

**DOI:** 10.1002/1878-0261.70169

**Published:** 2025-12-02

**Authors:** Silvia Eller, Susanne Ebner, Carmen Haselrieder, Julia K. Günther, Astrid Drasche, Sophie Strich, Chiara Volani, Andrea Medici, Aleksandar Nikolajevic, Alex Deltedesco, Johannes E. Sigmund, Michael J. Blumer, Martin Hermann, Johanna Vanacker, Gerald Brandacher, Eduard Stefan, Omar Torres‐Quesada, Jakob Troppmair

**Affiliations:** ^1^ Daniel Swarovski Research Laboratory, Department of Visceral, Transplant and Thoracic Surgery Medical University of Innsbruck Austria; ^2^ Institute of Molecular Biology and Center for Molecular Biosciences Innsbruck (CMBI) University of Innsbruck Austria; ^3^ Tyrolean Cancer Research Institute (TKFI) Innsbruck Austria; ^4^ Deparment of Internal Medicine II Medical University of Innsbruck Austria; ^5^ Department of Anatomy, Histology and Embryology, Institute of Clinical and Functional Anatomy Medical University of Innsbruck Austria; ^6^ Department of Anaesthesia and Intensive Care Medicine Medical University of Innsbruck Austria; ^7^ Division of Medical Biochemistry, Biocenter Medical University of Innsbruck Austria

**Keywords:** BRAFV600E, dabrafenib resistance, high‐resolution respirometry, melanoma, mitochondria, p66Shc

## Abstract

The emergence of resistance to mutant BRAF‐specific inhibitors (BRAFi) requires novel strategies for melanoma treatment. The progression of these tumors involves metabolic adaptations, which also affect the cellular redox status. Previous studies have linked RAF kinase signaling, a key component of the MAPK/ERK pathway involved in cell division and survival, to the suppression of mitochondrial reactive oxygen species (ROS) production, resulting in protection against cell death. In BRAF‐transformed cells, we have identified impaired JNK1/2‐dependent activation of the mitochondrial prooxidant protein p66Shc as a potential cause. In the present study, we dissected signaling and mitochondrial alterations that characterize the transition from BRAFi responsiveness to resistance in A375 melanoma cells. Insensitivity to BRAFi dabrafenib exposure was associated with reactivation of ERK1/2 phosphorylation, increased JNK1/2 kinase activity, p66ShcS36 phosphorylation, and elevated ROS production. Utilizing high‐resolution respirometry (HRR) and transmission electron microscopy (TEM), we show that dabrafenib‐resistant cells displayed mitochondrial damage, compensated by increased respiration, leading to high ROS levels. Moreover, dabrafenib‐resistant cells (A375D) have more efficient antioxidant systems, which may explain why despite ongoing cell death, net cell growth was observed. Treatment of both parental and resistant cells with phenethyl isothiocyanate (PEITC) increased ROS production but caused substantial cell death only in A375D melanoma cells. This PEITC effect could be demonstrated in two further dabrafenib‐resistant cell lines, WM164D and 451LuP. These results suggest that the altered redox status is linked to compromised mitochondria and is associated with the development of BRAFi resistance, rendering cells exquisitely sensitive to the actions of selective ROS‐inducing therapeutics.

## Graphical abstract

We show that dabrafenib‐resistant melanoma cells undergo mitochondrial remodeling, leading to elevated respiration and ROS production balanced by stronger antioxidant defenses. This altered redox state promotes survival despite mitochondrial damage but renders resistant cells highly vulnerable to ROS‐inducing compounds such as PEITC, highlighting redox imbalance as an Achilles' heel in BRAFi‐resistant melanoma.

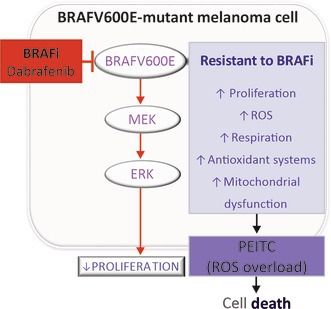

**Abbreviations**
A375D: A375 dabrafenib‐resistant
A375P: A375 parental
Ama: Antimycin A
AOi: Antioxidant inhibitor
BRAFi: BRAF inhibitor
CM: Complete medium
D: ADP
Dig: Digitonin
DMSO: Dimethyl sulfoxide
E: ET capacity
ERK1/2: Extracellular signal‐regulated kinase ½
ET: Electron transfer pathway capacity
GS: Glutathione
GSK3: Glycogen synthase kinase 3
HRR: High‐resolution respirometry
JNK1/2: c‐Jun N‐terminal kinase 1/2
L: LEAK respiration
MEF: Mouse embryonic fibroblast
mtOM: Mitochondrial outer membrane
N: NADH pathway
NS: NADH + Succinate pathway
Omy: Oligomycin
P: Oxidative phosphorylation (OXPHOS)
PEITC: Phenethyl isothiocyanate
PGM: Pyruvate, glutamate, malate
R: ROUTINE respiration
ROS: Reactive oxygen species
Rot: Rotenone
ROX: Residual oxygen consumption
S: Succinate pathway
Shc: Src homology 2
SV40: Simian virus 40
Trx: Thioredoxin
U: Uncoupler CCCP

## 1 Introduction

Despite major advances in the molecular understanding and in the therapy of melanoma, the prognosis remains bleak, with a five‐year survival rate of 16% for patients with distant metastases [1]. Melanoma progression is a multistep process initiated by an activating BRAF mutation already present in benign nevi. Subsequent mutations affecting cell cycle regulators and chromatin‐remodeling proteins (e.g., p53, PTEN) hyperactivate key intracellular signaling pathways, including MAPK (RAS‐RAF‐MEK‐ERK), PI3K, and mTOR [2]. Treatment with mutant BRAF‐specific inhibitors (BRAFi) initially showed promise, but recurrence is common, marked by the reactivation of ERK1/2 signaling [3]. Combining BRAF and MEK inhibitors provides improved outcomes, yet resistance inevitably develops [4]. The integration of checkpoint inhibitors is emerging as a potential preferred therapy for BRAFV600E mutated melanoma [5].

Oncogenic BRAF impacts cellular transformation through its effects on cell cycle progression, cell survival and in addition via metabolic reprogramming, characterized in melanoma by decreased oxidative phosphorylation (OXPHOS) and increased glycolysis [6]. BRAFi treatment decreases glycolytic activity, compensated by increased oxidative phosphorylation and elevated ROS production, a phenomenon observed in both responsive and resistant BRAF‐mutant melanoma cells [7, 8]. Resistant melanomas are characterized by increased oxidative metabolism and heightened dependence on glutamine over glucose [6].

p66Shc, the longest isoform of the ShcA family proteins, possesses a unique N‐terminal domain (CH2) [9, 10]. While the smaller isoforms mainly function in growth factor receptor signaling p66Shc is involved in mitochondrial prooxidant and pro‐death signaling [11]. Activation of p66Shc requires JNK1/2‐dependent phosphorylation of serine 36 (S36), followed by that of three additional residues by PKCß [12, 13, 14]. p66Shc has been linked to increased cellular redox stress under multiple pathological conditions including cancer [15]. A link between RAF signaling and p66Shc has been established by previously published observations: (i) We showed that signaling through RAF–MEK–ERK protected against mitochondrial ROS/Ca^2+^ accumulation and cell death [16, 17]. Consistently, lower ROS levels compared to other solid tumors have been reported for melanoma [18, 19], (ii) Phosphorylation of p66ShcS36 by JNK1/2 is compromised in BRAF‐transformed cells [20] possibly explaining reduced prooxidant stress in these cells.

Despite altered ROS levels, cancer cells remain sensitive to redox stress, and increasing ROS levels is a strategy employed in various treatment modalities, including mutant oncogenic kinase‐specific inhibitors [21, 22, 23]. In this study, we investigated the signaling and metabolic adaptations, that characterize the development of dabrafenib resistance in melanoma cells and possible effects on p66Shc signaling. Our data point to a deregulated ROS homeostasis resulting in increased ROS levels which drive tumor cell growth. Dabrafenib‐resistant cells become exquisitely sensitive to further increased ROS levels thereby opening a possible novel approach to their elimination.

## 2 Material and methods

### 2.1 Generation of dabrafenib‐resistant cell lines

Human melanoma cell lines A375 (RRID:CVCL_0132; ATCC, CRL‐1619), WM164 (RRID:CVCL_0217; Rockland Immunochemicals, Pottstown, PA, USA), and 451Lu (RRID:CVCL_6357; Rockland Immunochemicals) were maintained in Dulbecco's Modified Eagle Medium (DMEM; Lonza, Basel, Switzerland) supplemented with 10% (v/v) fetal bovine serum (FBS, Merck, Rahway, NJ, USA), 200 mm L‐glutamine, and 1% penicillin (100 U·mL^−1^)–streptomycin (100 μg·mL^−1^) (all from Gibco, Thermo Fisher Scientific, Waltham, MA, USA), hereafter referred to as complete medium, under standard growth conditions (37 °C, 5% CO_2_). Cells were routinely passaged at ~70–80% confluency to maintain optimal growth conditions. These melanoma cell lines were used to generate dabrafenib‐resistant sublines (A375D, WM164D, and 451LuD). Resistance was induced by exposing cells to escalating dabrafenib [(S2807, Selleckchem; Selleckchem, Housten, TX, USA) also known as GSK2118436] concentrations over a 16‐week period, aiming to achieve resistance to the clinically relevant plasma peak concentration of 1.5 μm dabrafenib [24, 25]. Therefore, 3 × 10^5^ cells were initially seeded in small flasks (25 cm^2^) containing 5 mL complete medium. The dosage started at 0.1 nm in the first week, followed by 1 nm in the second week, 10 nm in the third and fourth weeks, and escalated further to 100 nm in the fifth and sixth weeks. Subsequently, the dosage was increased to 300 nm in weeks 9 and 10, followed by 600 nm in weeks 11 and 12, and further elevated to 1000 nm in weeks 13 and 14. Finally, the dosage reached 1500 nm (1.5 μm) in the last 2 weeks. Throughout this process, the effect of dabrafenib treatment on cell viability was monitored. Following the acquisition of the resistant phenotype, cells were maintained in complete medium with weekly supplementation of 1.5 μm dabrafenib. As a control corresponding parental cell lines (A375P, WM164P, and 451LuP) were generated by subjecting them to the same treatment schedule using correlating concentrations of dimethyl sulfoxide (DMSO), the solvent used for dabrafenib. All cell lines, and their resistant sublines were routinely tested for mycoplasma contamination using the Mycoplasma PCR Kit (G238, abm, Richmond, BC, Canada) and confirmed to be mycoplasma‐free. The parental cell lines (A375, WM164, and 451Lu) were newly purchased from ATCC and Rockland at the beginning of the study and were not further authenticated.

### 2.2 Protein extraction and immunoblotting analysis

Cells were rinsed with PBS to remove residual culture medium and trypsinization was performed to detach cells from the culture surface. The cell suspension, combined with the previously collected culture medium, was centrifuged at 425 ***g*** for 10 min at 4 °C. The supernatant was discarded, and the cell pellet was washed with PBS, followed by centrifugation at 425 ***g*** for 10 min at 4 °C. After discarding the supernatant, protein extraction was initiated by adding RIPA Lysis and Extraction Buffer (89900; Thermo Scientific, Waltham, MA, USA) supplemented with protease (cOmplete™ Mini Protease Inhibitor Cocktail, Merck, Rahway, NJ, USA) and phosphatase inhibitor (PhosSTOP™, Merck, Rahway, NJ, USA), followed by incubation on ice with gentle shaking for 10–15 min. Subsequently, a final centrifugation step was performed for 10 min at 13 200 ***g*** and 4 °C. The resulting supernatant, containing the extracted proteins, was transferred to a fresh tube, and protein concentration was quantified using the Bio‐Rad, Hercules, CA, USA, DC protein assay kit (5000111; Bio‐Rad). Following quantification, an appropriate volume of 6× Laemmli buffer was added, and the mixture was heated at 95 °C for 5 min. Total protein ranging from 30 μg to 60 μg was separated on 7.5%, 10%, or 12.5% SDS/PAGE gels and subsequently transferred to nitrocellulose membranes as described previously [12, 13]. The following antibodies were employed for immunoblotting analyses: pERK1/2 (4377; Cell Signaling Technology, Danvers, MA, USA), ERK1/2 (9102; Cell Signaling Technology), pJNK1/2 (9251; Cell Signaling Technology), JNK1/2 (9252; Cell Signaling Technology), p66ShcS36 (ALX‐804‐358‐C100; Enzo, Farmingdale, NY, USA), Shc1 (610 082, BD Biosciences, Franklin Lakes, NJ, USA), pGSK‐3α/β (Ser21/9) (9331; Cell Signaling Technology), GSK‐3α (9338; Cell Signaling Technology), pHistone γH2A.X (2577; Cell Signaling Technology), PARP (9542; Cell Signaling Technology), and Anti‐Tubulin hFAB Rhodamine Antibody (12004164; Bio‐Rad). Secondary antibody detection involved the use of fluorescence‐labeled secondary antibodies, including Goat Anti‐Mouse IgG StarBright Blue 520 (12005866; Bio‐Rad), Goat Anti‐Mouse IgG StarBright Blue 700 (12004158; Bio‐Rad), Goat Anti‐Rabbit IgG StarBright Blue 520 (12005869; Bio‐Rad), Goat Anti‐Rabbit IgG StarBright Blue 700 (12004161; Bio‐Rad), Goat anti‐Rabbit IgG DyLight 800 (SA535571; Invitrogen, Carlsbad, CA, USA). The visualization was performed using the ChemiDoc Imaging System (Bio‐Rad), and protein expression was quantified with the Image Lab 5.2.1 Software (Bio‐Rad Laboratories).

### 2.3 High‐resolution respirometry

To analyze the mitochondrial function of A375P and A375D cells, an Oxygraph‐2 k‐FluoRespirometer (O2k, Oroboros Instruments; Innsbruck, Austria) device was used to measure O_2_ concentration and Amplex UltraRed fluorescence in real time. A375P cells were treated 3 h prior to the experiment with 1.5 μm dabrafenib. Cells were trypsinized and resuspended with a cell concentration of 2 × 10^6^ cells·mL^−1^ in mitochondrial respiration medium (MiR05; Oroboros Instruments), containing the same concentration of dabrafenib or solvent (DMSO) used during culture or treatment. The experiments were performed under constant stirring (750 rpm) and at 37 °C. The devices record oxygen concentration [μm] and oxygen flux [amol•s^−1^•x^−1^]. The unit “x” represents a single individual cell. Measurements were exclusively performed in MiR05 and therefore the O_2_ solubility factor of 0.92 was applied [26]. Instrumental O_2_ background tests were performed monthly. For the hydrogen peroxide (H_2_O_2_) flux analyses the fluorescence is measured with the Smart Fluo‐Sensors Green (Oroboros Instruments) with excitation at 525 nm and emission at ~600 nm [27]. The gain for the fluorescence sensor was set at 1000, which converts the amperometric raw signal of 1 μA into 1 V, and the fluorescence intensity was set at 500. The O2k device records H_2_O_2_ raw [V] and H_2_O_2_ slope [mV/s]. After O_2_ calibration, Amplex UltraRed (AmR) calibration is performed to determine fluorescence levels in the absence of the biological samples. First, 15 μm of the iron chelator diethylenetriamine‐N,N,N′,N,N‐pentaacetic acid (DTPA) is added to each chamber, to reduce background fluorescence slope of the AmR assay. Then, superoxide dismutase (SOD; 5 U·mL^−1^) and horseradish peroxide (HRP; 1 U·mL^−1^) are added before AmR (10 μm). Calibration of the fluorescence intensity emitted by the AmR assay is done after two titrations of hydrogen peroxide (H_2_O_2_; 0.1 μm). During the experiment multiple H_2_O_2_ calibration steps at different respiratory states need to be performed to quantify the sensitivity of the AmR assay over time. For the respiratory measurements, two substrate‐uncoupler‐inhibitor protocols (SUIT) were used to measure ROS fluxes and O_2_ consumption respectively. For simultaneous measurements of H_2_O_2_ and O_2_ fluxes, we generated a SUIT protocol optimized for A375 cells, the DLPu O_2_ ce‐pce AmR. The biological samples were put into the chambers by full replacement. The protocol starts with the titration of 15 μm DTPA, followed by stirring for 10 to 15 min. It continues with the addition of 5 U·mL^−1^ SOD, 1 U·mL^−1^ HRP and 10 μm AmR. Afterwards, ROUTINE state (*R*) can be measured. In the next step, O_2_ concentration is reduced to 60 μm through N_2_ gas addition and the plasma membrane is permeabilized by the addition of the optimum effective digitonin (Dig) concentration. In a previous experiment the optimal concentration of 10 μg·mL^−1^ digitonin for A375P and 5 μg·mL^−1^ for A375D cells was determined. Subsequently, the first H_2_O_2_ (0.1 μm) calibration step is performed. Next, pyruvate (P; 5 mm), glutamate (G; 10 mm), and malate (M; 2 mm) are added to the chambers to induce LEAK (*L*). This step is followed by the addition of the antioxidant inhibitors (AOi). First, DNCB is added in multiple titration steps, to avoid ramping in the H_2_O_2_ flux. Second, auranofin is added in a single titration step. Afterwards, the OXPHOS state (*P*) is induced by adding ADP (D; 2.5 mm) in kinetically saturating concentrations. It is necessary to wait for around 10 min until the O_2_ flux is in a steady state. In the next step another H_2_O_2_ (0.1 μm) calibration is performed. Subsequently, 10 mm succinate is added followed by 10 nm Oligomycin (Omy). The protocol continues with the addition of 0.5 μm rotenone (Rot) and H_2_O_2_ calibration (0.1 μm), followed by the titration of 2.5 μm antimycin A (Ama) and a final H_2_O_2_ (0.1 μm) calibration step. Finally, to assess coupling control and membrane integrity in these cells, we used the SUIT‐008 O2 ce‐pce D025 protocol [26]. In a similar way, cells were placed in the chambers by complete volume replacement. First, ROUTINE respiration (*R*) is measured. In the next step, the plasma membrane is permeabilized by 5–10 μg·mL^−1^ digitonin. Thereafter, LEAK (*L*) is induced by the addition of pyruvate (5 mm) and malate (2 mm). To reach the active OXPHOS state (*P*) 2.5 mm ADP is added. Afterwards, 10 μm cytochrome c (c) is added, followed by the addition of 10 mm glutamate. By adding 10 mm succinate, the convergent electron flow in the NADH succinate pathway is stimulated. Subsequently, stepwise CCCP uncoupler (U) titrations (0.5 or 1 μm/step) follow until the maximum in the oxygen flux is reached to achieve the maximum electron transfer capacity (ET, *E*). At the end, 0.5 μm rotenone and 2.5 μm antimycin A are added. All O2k data analyses were performed with the DatLab 7.4 software. Respiratory rates were corrected for the instrumental O_2_ background flux, dilution of the sample by titrations, and residual oxygen consumption (ROX).

### 2.4 Mitochondrial membrane potential measurements (TMRM)

Mitochondrial membrane potential (∆Ψm) was assessed on living cells using the potentiometric fluorescent dye tetramethyl rhodamine methyl ester (TMRM, Invitrogen I34361). Cells were seeded on ibidi 8‐well chamber slides (ibidi, 80806, ibidi GmbH, Gräfelfing, Germany) at a cell density of 30.000 cells/cm^2^. On the following day, prior to the TMRM analysis, living cells were incubated for 1 h in Opti‐MEM (31985070; Gibco). Next, cells were stained with Opti‐MEM containing 20 nm TMRM for 30 min in the dark at 37 °C. Immunofluorescence analysis was performed using an Olympus Microscope (IX83 P2ZF) with a UPLSAPO 2 10×/0.4 objective. The accumulation of TMRM at the mitochondria was quantified as mean gray intensity of the red light using the Neuronal Network system provided by the software OLYMPUS cellSens Dimension 4.4.

### 2.5 Transmission electron microscopy (TEM)

A375P and A375D cells were fixed in 2.5% glutaraldehyde (GA) and 2% PFA in 0.1 m sodium cacodylate buffer (pH = 7.4) for 1 week at 4 °C. Subsequently, they were rinsed in sodium cacodylate buffer and post‐fixed in 0.5% osmium tetroxide and 1% potassium ferricyanide in distilled water overnight at 4 °C. Samples were rinsed again, dehydrated in a graded ethanol series and acetone and embedded in EPON resin (45359; Sigma‐Aldrich, St. Louis, MO, USA). Ultrathin sections (90 nm) were cut on a Reichert Ultracut S microtome (Leica Microsystem, Wetzlar, Germany) with an ultradiamond knife (Diatome, Biel, Switzerland) and mounted on dioxane formvar‐coated slot grids (PYSL2010S‐CU, Science Services, München, Germany). The sections were examined at 80 kV with a Philips CM 120 transmission electron microscope (FEI, Eindhoven, The Netherlands) equipped with a MORADA digital camera and iTEM software (Olympus SIS, Münster, Germany).

### 2.6 Annexin V/7AAD staining after PEITC stimulation

To evaluate PEITC (253731, Sigma‐Aldrich) induced cell death and the potential protective effect of the mitochondrial antioxidant MitoTempo (HY‐112879, MedChemExpress, Monmouth Junction, NJ, USA), Annexin V/7AAD staining followed by flow cytometric analysis was performed. For the concentration‐dependent experiments, A375P and A375D cells were seeded at 3 × 10^5^ and 4 × 10^5^ cells, respectively, in T25 flasks and incubated for 48 h in complete medium. Cells were then treated with increasing concentrations of PEITC (0 μm, 5 μm, 15 μm, or 30 μm) for 30 min at 37 °C. In additional experiments, A375, WM164, and 451Lu cells (parental and resistant) were seeded at 4 × 10^5^ cells per well in 6‐well plates and incubated for 24 h. Cells were subsequently treated with 20 μm PEITC for cell line–specific durations: 30 min (A375), 1.5 h (WM164), or 2 h (451Lu), with or without a 16‐h pretreatment with 20 μm MitoTempo. Following treatment, both adherent and floating cells were harvested and stained using the Annexin V/7AAD Apoptosis Detection Kit (640 922, BioLegend, San Diego, CA, USA) according to the manufacturer's instructions. Flow cytometric analysis was performed using an LSRFortessa™ cytometer (BD Biosciences), and data were analyzed with FlowJo™ software (version 10.7).

### 2.7 ROS measurement

Mitochondrial ROS levels were visualized through fluorescence microscopy, employing a staining protocol with 100 nm MitoTracker Red CM‐H2XROS (M46753; Thermo Fisher Scientific) in serum‐free DMEM, following the manufacturer's guidelines. 2 × 10^4^–2.5 × 10^4^ A375P and A375D cells were seeded in 8‐well LabTek chamber slides and cultured for 24 h in complete medium. Some cell populations were exposed to 20 μm MitoTempo for 16 h to modulate baseline ROS levels. ROS staining, lasting 25 min at 37 °C, was conducted under two conditions: unstressed and stressed, the latter induced by treating the cells with 20 μm PEITC during the staining period. Digital images of the cells were captured using an Olympus IX70 inverted microscope with a 40× water immersion objective (numerical aperture 0.8) and an Olympus U‐RFL‐T mercury‐vapor lamp. Image acquisition utilized the Olympus UC90 camera and Olympus CellSens software 3.1, employing a 568‐nm filter for MitoTracker Red CM‐H2XROS. Gray values were quantified using Fiji/Image J software, averaging the values from 50 cells across at least four different images for each experimental condition.

### 2.8 Statistical analysis

Statistical analysis was conducted using GraphPad Prism 9.4.1 software (GraphPad, La Jolla, CA, USA). Prior to applying parametric statistical tests, we assessed the assumption of normality using the Shapiro–Wilk test. Depending on whether the data followed a normal distribution or not, we employed ANOVA with Dunnett's post hoc analysis or the Kruskal–Wallis test with Dunn's post hoc analysis for multiple comparisons, respectively. For comparisons involving two groups, we applied either the *t*‐test or Wilcoxon test for paired datasets, and the *t*‐test or Mann–Whitney test for unpaired datasets, depending on data distribution. The levels of significance were *P* < 0.05 (*), *P* < 0.01 (**), *P* < 0.001 (***), and *P* < 0.001 0.0001 (****).

## 3 Results

### 3.1 Growth characteristics and signaling adaptations in dabrafenib‐resistant A375 melanoma cells

**Fig. 1:**
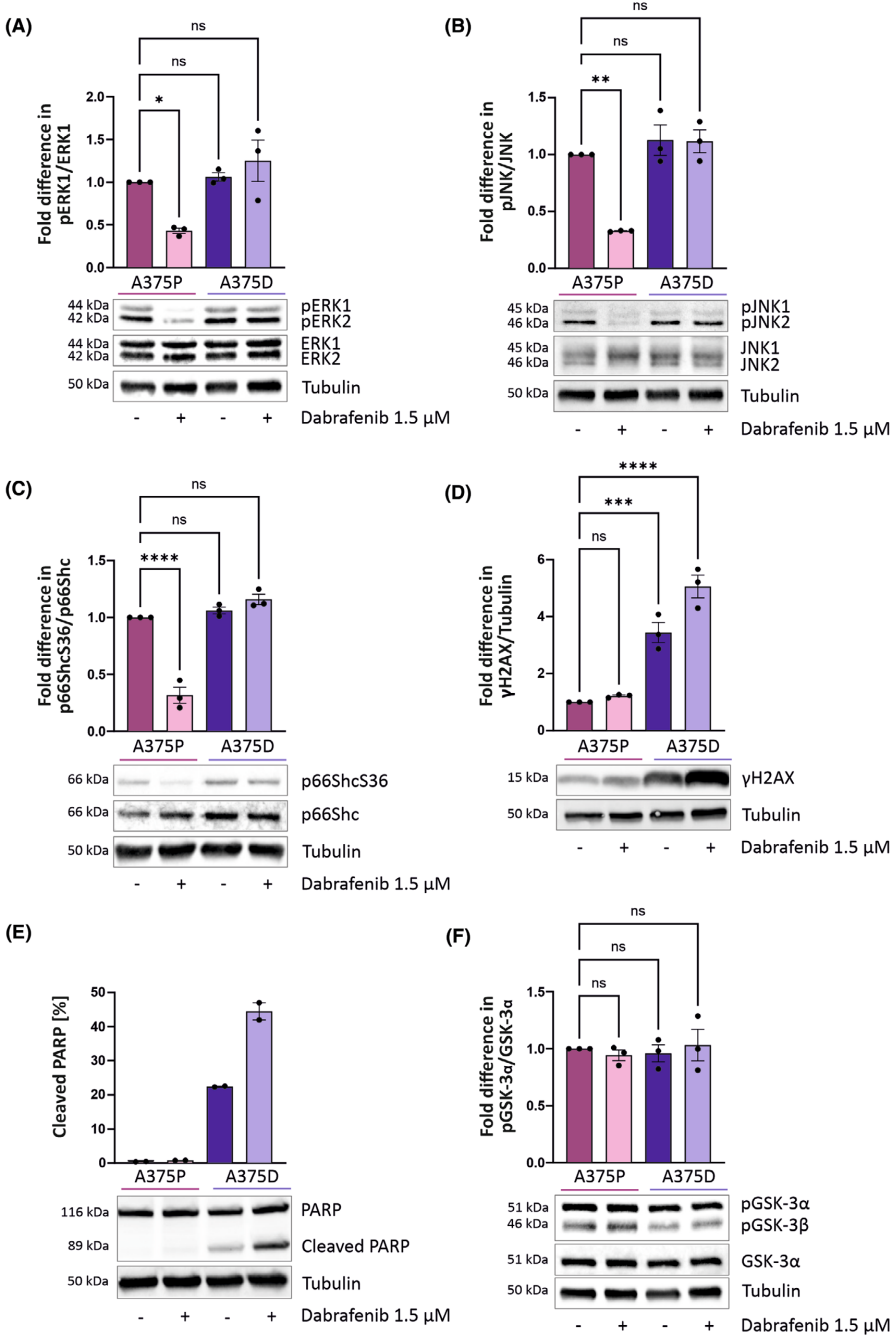
Effect of dabrafenib on intracellular signaling in A375P and A375D cells. Cells were treated with 1.5 μm dabrafenib for 3 h prior to lysis. Immunoblots were carried out with antibodies specific to phosphorylated and total (A) ERK, (B) JNK, (C) p66ShcS36, and (F) GSK3. Immunoblots with antibodies specific for (D) DNA damage γH2AX (S39) and (E) apoptosis marker PARP (shown as a percentage of cleaved PARP) were also conducted. Data are presented as mean ± SEM. Statistically significant differences compared to the untreated A375P group were tested using ordinary one‐way ANOVA; *N* = 3 (except PARP, *N* = 2). (*P ≤ 0.05; ** *P* ≤ 0.01; *** *P* ≤ 0.001 and ****P ≤ 0.0001). Uncropped gel images are shown in Fig. S9.

Following the generation of parental (A375P) and dabrafenib‐resistant (A375D) melanoma cells, we characterized their viability under increasing concentrations of dabrafenib to confirm their phenotype. As shown in Fig. S1A–C, A375P cells were sensitive to dabrafenib, marked by growth arrest and the onset of cell death across a broad concentration range. In line with expectations, A375P cells exhibited constitutive ERK1/2 phosphorylation due to the upstream BRAF mutation, which decreased upon dabrafenib treatment (Fig. 1A). In contrast, A375D cells displayed elevated ERK1/2 activity that was unresponsive to dabrafenib (Fig. 1A).

In previous work, we showed that partial knockdown of p66Shc revealed its tumor‐suppressive potential in BRAFV600E‐transformed NIH3T3 fibroblasts and in A375 cells carrying the same mutation. Because p66Shc activation—measured by phosphorylation at serine 36 (S36)—was shown to depend on JNK1/2 [12], we next examined JNK1/2 signaling in A375P and A375D cells. JNK1/2 activity detected in A375P cells decreased upon BRAF inhibition but remained unchanged in resistant cells (Fig. 1B).

A375D cells showed increased p66Shc levels (Fig. 1C). Phosphorylation of p66Shc at S36 was low in A375P cells and further decreased with dabrafenib treatment (Fig. 1C), suggesting BRAF involvement in JNK1/2 activation and phosphorylation of p66Shc at S36. Notably, phosphorylation of p66Shc at S36 is required for its prooxidant activity, leading to H_2_O_2_ production through mechanisms that are still under debate [11, 28].

γH2AX serves as a well‐established marker for DNA damage and it is strongly linked with elevated ROS levels [29]. As depicted in Fig. 1D, the acquisition of dabrafenib resistance led to markedly increased γH2AX levels, indicating enhanced DNA damage response and suggesting increased ROS production in A375D cells. ROS levels have been linked to the induction of cell death through both p66Shc‐dependent [9, 30, 31] and p66Shc‐independent [32, 33, 34, 35, 36] pathways. Cleavage of the caspase substrate PARP showed that PARP processing in resistant cells under normal growth conditions was further enhanced by dabrafenib treatment (Fig. 1E). Finally, we included GSK3 as a BRAF‐independent downstream effector of the RAS–PI3K–AKT pathway to assess whether the resistance phenotype was confined to the MAPK axis or extended to other signaling branches [37]. As shown in Fig. 1F, GSK3 phosphorylation remained unchanged upon dabrafenib treatment in both A375P and A375D cells, suggesting that BRAFi resistance does not broadly alter AKT‐mediated signaling.

### 3.2 Metabolic alterations in BRAFi‐resistant melanoma cells

**Fig. 2:**
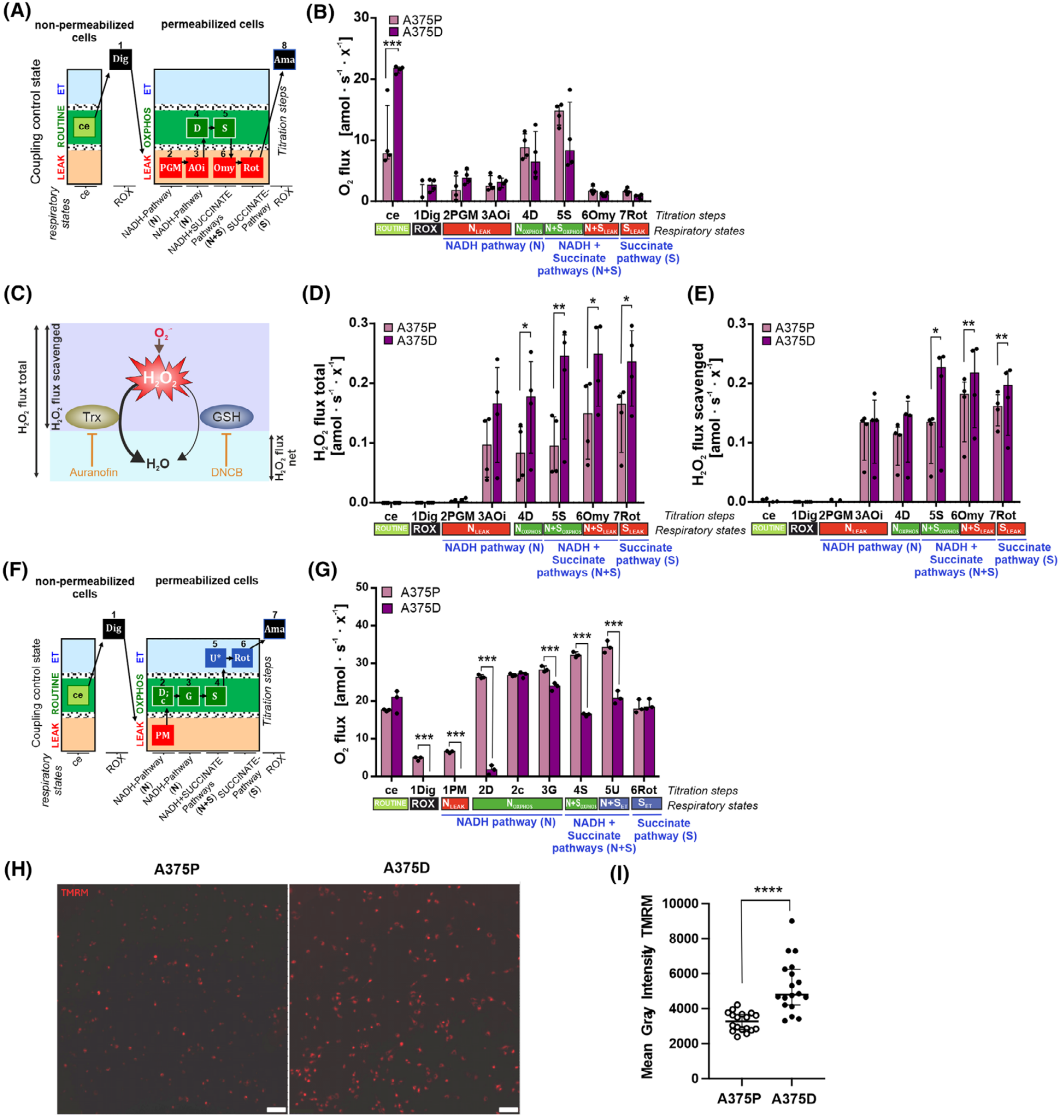
Dabrafenib resistance impacts mitochondrial function, including respiration, ROS production, and membrane potential, in A375 cells. (A) Substrate‐uncoupler‐inhibitor titration (SUIT) Protocol for the combined measurements of Amplex UltraRed H_2_O_2_ production (ROS) and oxygen consumption using high‐resolution respirometry (HRR) [26, 38]. Image adapted from [60]. (B) Oxygen fluxes [amol•s^−1^•x^−1^] baseline‐corrected for *Rox*, of A375P and A375D resistant cells. (C) Schematic depiction of the H_2_O_2_ production and scavenging of the Thioredoxin (TrX) and Glutathione (GSH) antioxidant systems in our system to measure ROS and antioxidant capacities. The inhibitors against both systems (Auranofin and DNCB) are shown. H_2_O_2_ fluxes scavenged for the antioxidant systems corrected for the background slope and *Rox* baseline of the combined respirometric measurements of B (D) H_2_O_2_ fluxes corrected for the background slope and *Rox* baseline of the combined respirometric measurements of B. (E) H_2_O_2_ fluxes scavenged for the antioxidant systems corrected for the background slope and *Rox* baseline of the combined respirometric measurements of D. (F) SUIT Protocol used for the respirometric measurements of oxygen consumption to assess ROUTINE, LEAK, OXPHOS, and ET (electron transfer pathway capacity) using HRR [26]. Image adapted from [61]. (G) Oxygen fluxes [amol•s^−1^•x^−1^] baseline‐corrected for *Rox*, with P, M and G as substrates for the NADH pathway, cytochrome *c* for integrity of the mtOM, and S for the Succinate pathway of A375P, A375P and A375D resistant cells. Ama, antimycin A; AOi, antioxidant system inhibitors (DNCB 2.5 μm, Auranofin 120 nm); D, ADP; Dig, digitonin; *E*, ET capacity; *L*, LEAK; N, NADH electron transfer‐pathway state; NS, NS‐pathway; Omy, oligomycin; *P*, OXPHOS; PGM, pyruvate, glutamate, malate; *R*, ROUTINE respiration; Rot, rotenone; ROX, residual oxygen consumption; S, Succinate pathway; S, succinate; U, uncoupler CCCP. Results from B, D, E, and G are represented as median ± IQR (50% range). 2‐way ANOVA with Bonferroni's multiple comparison test or nonparametric unpaired *t*‐test analysis; *N* = 4. (* *P* ≤ 0.05; ** *P* ≤ 0.01; *** *P* ≤ 0.001). (H) Representative images of the TMRM assay; scale bar = 100 μm. (I) Quantification of the TMRM signal in A375P and A375D cells. Data were background corrected. Data presented as Median with 95% CI. *N* = 4. *P*‐value < 0.001 using a Mann–Whitney nonparametric test.

To investigate how dabrafenib resistance affects mitochondrial function and reactive oxygen species (ROS) production, we analyzed oxygen and hydrogen peroxide (H_2_O_2_) fluxes in both parental A375P and resistant A375D melanoma cells using high‐resolution respirometry. For a comprehensive assessment of ROS generation in relation to mitochondrial activity, we performed simultaneous measurements of H_2_O_2_ fluxes and oxygen consumption. To profile the contribution of the different metabolic substrates to mitochondrial function and ROS production, we performed sequential titrations of the respiratory substrates and inhibitors using a substrate‐uncoupler‐inhibitor titration (SUIT) protocol [26], specifically designed for this study (Fig. 2A) [26]. This protocol combines the major NADH‐linked substrates (pyruvate, glutamate, and malate) with succinate under LEAK conditions—which refers to the mitochondrial oxygen consumption that occurs due to proton leak, representing a non‐phosphorylating resting state—and it is known to be the respiratory state most associated with ROS production [38]. Moreover, to avoid interference from the antioxidant systems on H_2_O_2_ levels and to analyze the contribution of those systems to BRAFi resistance, we used inhibitors against the major antioxidant systems, DNCB against Glutathione (GSH) and Auranofin against the Thioredoxin (Trx) systems.

First, our analysis showed A375D cells have a different mitochondrial bioenergetic profile than A375P cells. Specifically, the mitochondrial ROUTINE respiration—which reflects steady state respiration linked to cellular energy demand—was higher in the dabrafenib‐resistant A375D cells compared to the parental A375P cells (Fig. 2B). This observation was confirmed using a protocol where the A375 and, additionally, WM164 cells were not permeabilized and were measured in the presence of cell culture media, revealing that resistant cells show enhanced mitochondrial respiration (Fig. S2A,D). Furthermore, A375D cells show a nonsignificant increase in LEAK respiration (Fig. 2B), suggesting that A375D cells display a dyscoupling related to mitochondrial dysfunction, which was supported in the experiments using non‐permeabilized cells by a reduction in the ROUTINE/ET (*R/E*) ratio. The *R/E* ratio serves as an indicator of respiratory efficiency—lower values suggest reduced utilization of the electron transfer capacity, which may result from either increased respiratory reserve or mitochondrial dysfunction [26]. Furthermore, we performed bioenergetic cluster analysis of ROUTINE versus LEAK respiration to detect whether the A375P and A375D bioenergetic profiles differ, and results further support this observation, showing that resistant cells form a distinct cluster characterized by elevated LEAK respiration [39]. These findings suggest the existence of a mitochondrial dysfunction in A375D and WM164D cells (Fig. S2B,C,E,F). Additionally, antioxidant inhibitors suppressed respiration in A375P cells but had little effect on A375D cells (Fig. S3A,C,E,G), implying that the resistant cells may have adaptations in the GSH and Trx antioxidant systems.

Second, to assess whether ROS levels are influenced by drug resistance or dabrafenib treatment, we measured H_2_O_2_ production in both parental A375P and resistant A375D cells. Since H_2_O_2_ levels are strongly regulated by antioxidant activity and we could not detect ROS levels under steady state conditions, we calculated the H_2_O_2_ fluxes scavenged by the Trx and GSH systems. This allowed us to evaluate not only ROS emission but also the efficiency of these antioxidant systems (Fig. 2C). Our analysis revealed that A375D cells exhibited the highest H_2_O_2_ fluxes in the presence of antioxidant inhibitors (Fig. 2D), indicating elevated ROS production. Moreover, the higher scavenging activity found in A375D cells suggests that the Trx and GSH systems are more efficient in the resistant cells compared to the parental ones. H_2_O_2_ levels were almost undetectable in both A375D and A375P cells prior to the addition of the inhibitors Auranofin and DNCB (Fig. 2E, Fig. S3B,D,F,H).

Additionally, since our initial experiments revealed differences in mitochondrial respiration between A375P and A375D cells, we conducted an extended analysis to further characterize their mitochondrial bioenergetic profiles. Using the SUIT‐008 coupling control protocol [40] (Fig. 2F), we evaluated mitochondrial respiration in the respiratory states LEAK, OXPHOS, and capacity of the electron transfer system (ET), representing the non‐phosphorylating, phosphorylating, and maximum respiration, respectively. In addition, we assessed the integrity of the mitochondrial outer membrane (mtOM) and performed a comprehensive analysis of both the NADH‐linked (N‐pathway, which comprises NADH‐generating substrates feeding electrons through Complex I) and succinate‐linked (S‐pathway, which feeds electrons from succinate through Complex II) respiration at the OXPHOS and ET states (Fig. 2F, Fig. S3I) using specific substrate titrations [26]. Analysis of oxygen fluxes revealed significant damage to the mitochondrial outer membrane (mtOM) in A375D cells, as indicated by reduced cytochrome c control efficiency—an established marker of mitochondrial integrity (Fig. 2G, Fig. S3J). Additionally, the addition of glutamate inhibited respiration in A375D cells, suggesting a possible dysfunction in the glutamate pathway (Fig. 2G, Fig. S3K). This impairment is further corroborated by the low NADH/NADH+Succinate pathway control ratio (Fig. S3L), indicating weakened NADH‐linked respiration. In contrast, the Succinate/NADH+Succinate pathway control ratio was elevated (Fig. S3M), suggesting a compensatory activation of the succinate pathway.

To further investigate how dabrafenib resistance affects mitochondrial membrane potential (∆Ψm), we performed TMRM staining experiments. The results showed that A375D cells exhibit a higher TMRM signal compared to A375P cells, indicating an elevated mitochondrial membrane potential in the resistant cells (Fig. 2H,I). This finding is consistent with the increased respiration observed in dabrafenib‐resistant cells, suggesting a compensatory mechanism to maintain energy production despite mitochondrial dysfunction.

In summary, our data show that A375D resistant cells exhibit severe mitochondrial dysfunction, including mtOM damage and impaired glutamate metabolism. Despite this, they maintain higher overall respiratory activity and ROS production compared to parental cells, likely due to compensatory mechanisms and possible shifts in mitochondrial populations. These findings suggest that dabrafenib resistance is associated with mitochondrial remodeling, ultimately leading to increased oxidative phosphorylation capacity and higher ROS emission associated with mitochondrial dysfunction.

**Fig. 3:**
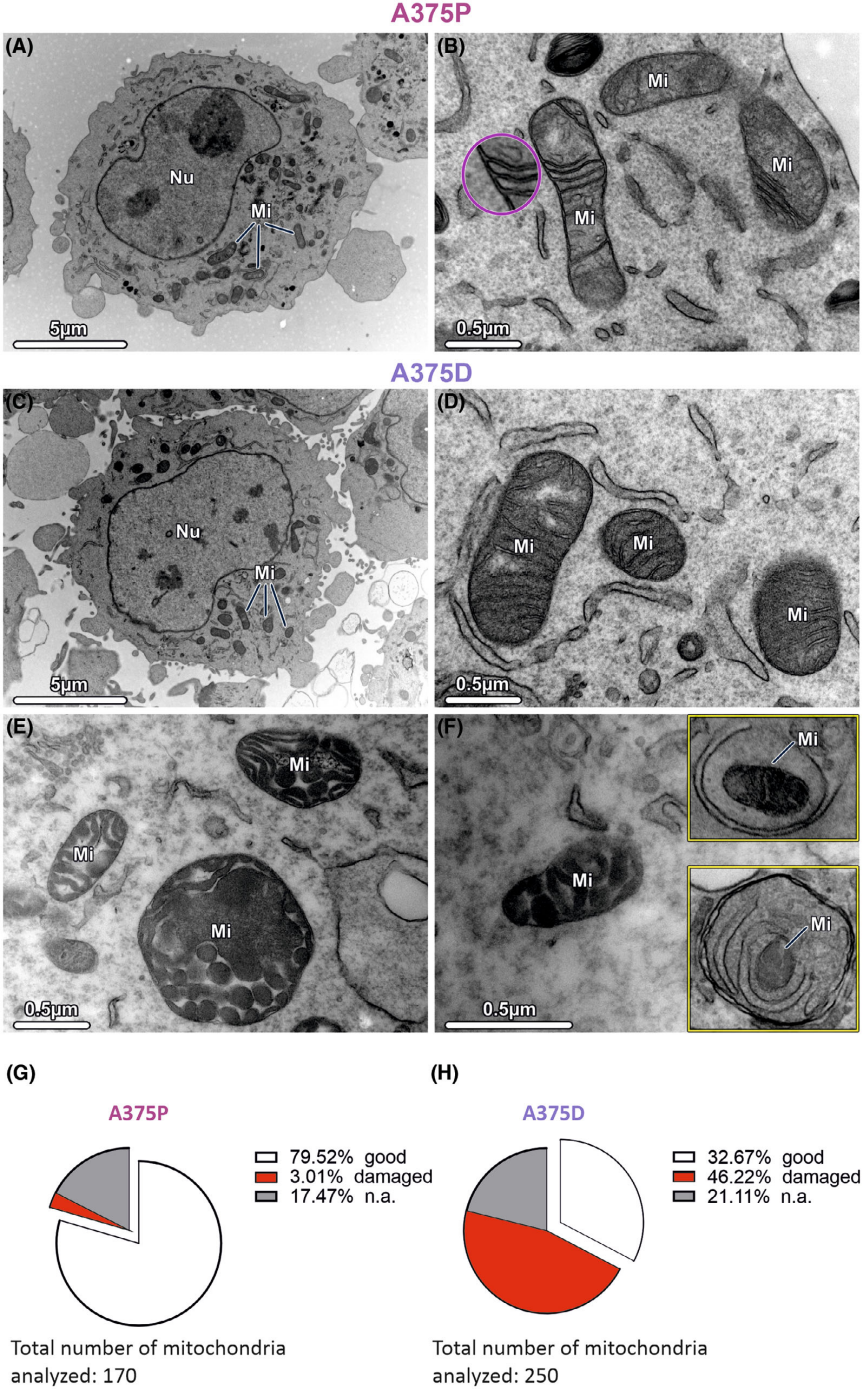
Mitochondrial ultrastructure is affected in A375D cells. Representative transmission electron micrographs of (A, B) A375P cells and (C–F) A375D cells depict differences in mitochondrial ultrastructure. Mitochondria with intact double membrane and inner membrane invaginations (cristae) were observed in both (B) A375P cells and (D) A375D cells. In contrast, altered mitochondria, characterized by a swollen appearance and damaged cristae, were specifically observed in (E) A375D cells. (F) Mitophagy was observed in A375D cells and is highlighted with yellow outlines. (G, H) Quantitative analysis of mitochondrial integrity. Mitochondria were classified as “good” (intact double membrane and cristae), “damaged” (swollen or fragmented mitochondria, with disrupted cristae or outer membrane), or “n.a.” (not assessable). A clear difference in the percentage of “good” and “damaged” mitochondria was found between the A375P and A375D cells, while the percentage of nonassessable mitochondria was almost the same in both groups. A total of (G) 170 mitochondria from A375P cells and (H) 250 mitochondria from A375D cells were analyzed. Pink circle shows magnification of the mitochondrial structure in B. Results represent pooled data from three independent experiments, expressed as percentages. Mi, Mitochondria; Nu, Nucleus. Scale bars = 5 μm/0.5 μm.

Finally, to investigate whether the mitochondrial functional profiles are related to changes in mitochondrial morphology, we also performed transmission electron microscopy (TEM) to detect changes in mitochondrial ultrastructure between A375P and A375D cells. In A375P cells, the vast majority of mitochondria exhibited a normal ultrastructure (Fig. 3A,B), whereas mitochondria in resilient cells (A375D) tended to undergo ultrastructural changes (Fig. 3C–F). These mitochondria were enlarged and exhibited a condensed morphology, with the double membrane and cristae not clearly visible (Fig. 3E,F). Moreover, we occasionally observed signs of mitophagy (Fig. 3F, insets). Remarkably, damaged and normal mitochondria were never found in the same cell, but several cells additionally contained mitochondria that could not be clearly assigned. Quantification revealed 46.22% of damaged mitochondria in the A375D cell line compared to 3.01% in the A375P cell line (Fig. 3G,H).

### 3.3 Targeting metabolic alterations overcomes dabrafenib resistance in melanoma cells

A375D cells exhibit a shift in mitochondrial metabolism that leads to increased production of ROS. While elevated ROS levels are a common feature of tumor cells [18], they must remain below a critical threshold to prevent the death of cancer cells while normal cells remain unaffected [22]. To explore whether further elevation of ROS could have different effects on parental and resistant melanoma cells, we tested three compounds known to affect mitochondrial ROS production: PEITC [41], MitoPQ—which induces superoxide at complex I of the electron transport system [42]—and the OXPHOS inhibitor IACS [43]. Notably, IACS does not induce ROS production in all cell lines [44]. IACS was ineffective in inducing cell death in A375 cells, while MitoPQ did not increase ROS levels (Fig. S4). We previously established a causal link between BRAFV600E‐driven transformation and increased ROS production via the JNK1/2‐p66Shc signaling axis [20]. In BRAFV600E‐transformed NIH3T3 fibroblasts as well as in the BRAFV600E‐mutated melanoma cell lines A375 and M238, we demonstrated that PEITC treatment led to increased ROS production. This effect was blocked by pretreatment with a JNK1/2 inhibitor. In BRAFV600E‐transformed NIH3T3 fibroblasts, we also demonstrated that shRNA‐mediated knockdown of p66Shc decreased ROS production and led to enhanced growth in soft agar. The same effect was observed in A375 cells [20].

**Fig. 4:**
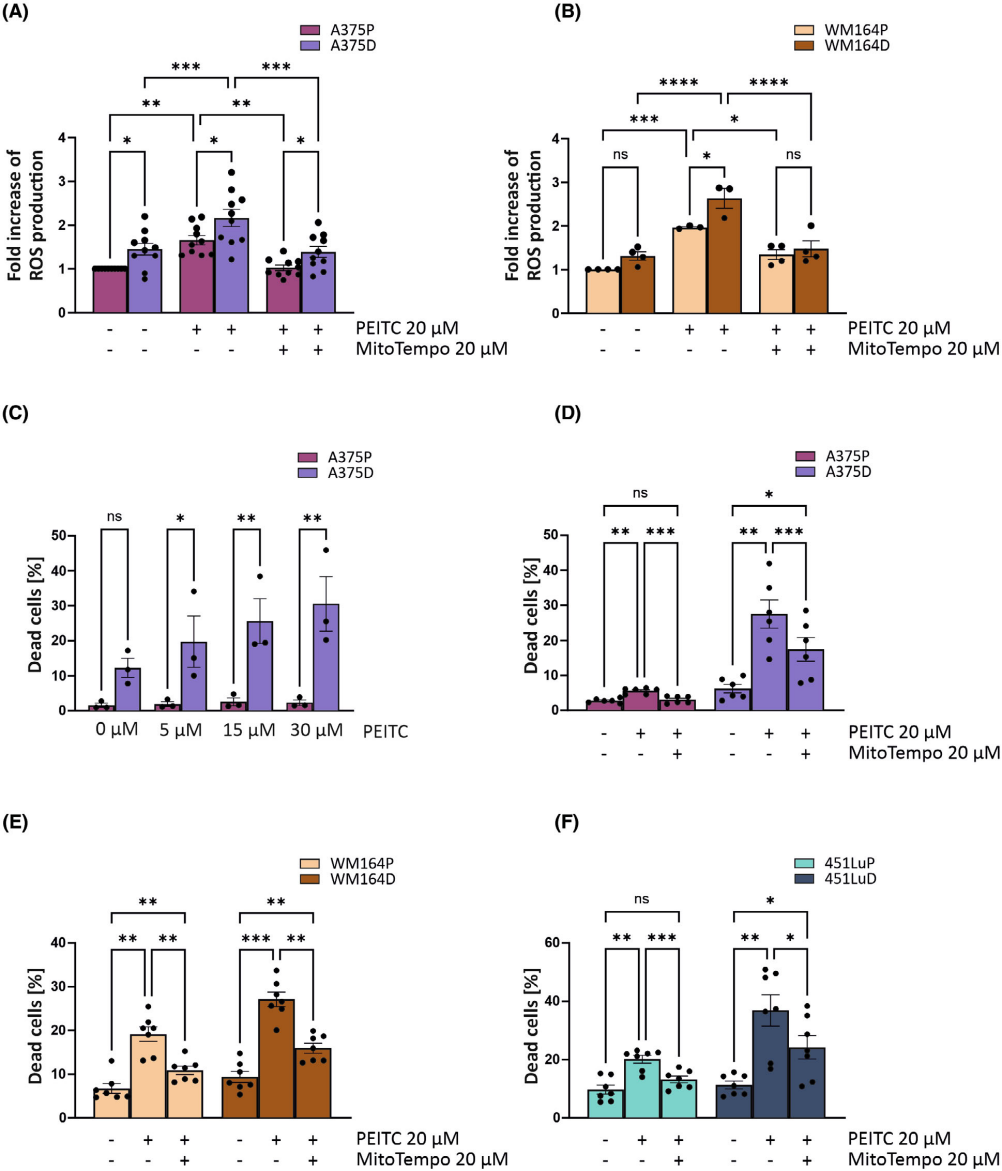
Overcoming resistance upon PEITC treatment. ROS levels were measured after a 30‐min treatment with 20 μm phenethyl isothiocyanate (PEITC), alone and in combination with a 16‐h pretreatment with the antioxidant MitoTempo. Mitochondrial ROS levels were assessed using MitoTracker Red CM‐H_2_XRos staining. Quantification of ROS levels is shown for (A) A375P and A375D cells (*N* = 10) and (B) WM164P and WM164D cells (*N* = 4). (C) Cell death was evaluated following treatment with increasing concentrations of PEITC (5 μm, 15 μm and 30 μm) for 30 min, as analyzed by flow cytometry using Annexin V/7AAD staining. The percentage of dead cells is displayed; *N* = 3. Cell death was further analyzed by flow cytometry after a 30 min treatment with PEITC in (D) A375P and A375D cells, a 1.5 h treatment with 20 μm PEITC in (E) WM164P and WM164D cells, and a 2 h treatment in (F) 451LuP and 451LuD, each alone or in combination with a 16 h pretreatment with the antioxidant MitoTempo; *N* = 7. Data are presented as Mean ± SEM. Statistically significant differences between groups were tested using an ordinary one‐way ANOVA with Holm–Šídák correction for multiple comparisons and ordinary one‐way ANOVA. (*P ≤ 0.05; **P ≤ 0.01; ***P ≤ 0.001).

In our current study, both A375P and A375D cells showed increased ROS production in response to PEITC treatment, which was quenched by pre‐incubation with the antioxidant MitoTempo (Fig. 4A). These observations were also confirmed in WM164 cells (Fig. 4B). Of note, PEITC selectively induced cell death in A375D cells while leaving A375P cells largely unaffected (Fig. 4C,D, Fig. S5), revealing a novel vulnerability of BRAFi‐resistant melanoma cells. To assess whether this effect is of broader relevance, we tested PEITC in two further independently established pairs of melanoma cells: WM164P/WM164D and 451LuP/451LuD (Fig. S6). Similar to A375D cells, WM164D and 451LuD also exhibited resistance to cell death and maintained constitutive ERK1/2 phosphorylation, which remained unaffected by dabrafenib. Upon acquisition of BRAFi resistance, both WM164D and 451LuD cells showed markedly increased sensitivity to PEITC treatment compared to their parental counterparts (Fig. 4E,F; Figs S7 and S8). In both cell lines, additional treatment with MitoTempo markedly, though not completely, reduced PEITC‐induced cell death (Fig. 4E,F), suggesting that ROS contribute to the cytotoxic effect.

## 4 Discussion

In the work presented here, we addressed metabolic alterations distinguishing parental from dabrafenib‐resistant melanoma cells and identified altered redox status as one major phenotype of the drug‐resistant cells. We were also able to show that elevated ROS levels present in these cells may provide a growth advantage; however, further increasing them by treatment with PEITC triggers cell death more readily in resistant than in parental cells.

Although early discoveries of mutations in oncogenes and tumor suppressor genes were primarily associated with the classical hallmarks of cancer—such as sustaining proliferative signaling, evading growth suppressors, resisting cell death, enabling replicative immortality, inducing angiogenesis, and promoting invasion and metastasis [45], tumor‐associated metabolic alterations have come into focus anew over the last decade as drivers of tumor progression and targets for therapeutic interference. Both, established cancer pathways and mutations in metabolic enzymes have been implicated in this metabolic rewiring [45]. In melanoma, BRAFi sensitivity has been found to correlate with the glycolytic response in preclinical studies [46] and clinical studies [47]. Treatment with BRAF inhibitors causes BRAF mutant melanoma cells to become dependent on oxidative phosphorylation (OXPHOS) [48], compensating for impaired glycolysis. Importantly, early findings from clinical trials using mitochondrial inhibitors such as biguanides have been mostly disappointing [49, 50]. Recent preclinical studies suggest that targeting the mechanisms driving metabolic plasticity and adaptation in cancer cells may provide a more promising therapeutic strategy [51].

Previously we showed for the first time that BRAF/CRAF signaling limited mitochondrial ROS levels under stress to prevent cell death [17]. This mechanism may be exploited in tumor treatment as BRAF/MEK inhibition increased ROS levels as predicted, which resulted in cell killing once the protective effect of Bcl‐2 was neutralized [21, 23]. We also described a defect in the activation of p66Shc‐dependent mitochondrial ROS production in BRAF‐transformed cells due to compromised JNK1/2 activation, which is required for p66Shc S36 phosphorylation, that is essential for the prooxidant/pro‐death function of p66Shc [20]. These findings demonstrate apart from other metabolic alterations, compromised ROS production may be a hallmark of BRAF transformation. Development of BRAFi resistance goes along with restoration of JNK1/2 signaling, activated p66Shc phosphorylation, increased ROS production and increased cell death rate. Efficient killing of these cells requires—as a further stimulus—the treatment with PEITC, which has been previously implicated in the p66Shc‐dependent killing of prostate cancer cells [52].

Mitochondrial function is deregulated in melanoma through various mechanisms. Mutations in the MAPK pathway activate the PI3K pathway, leading to the phosphorylation of phospholipid‐dependent kinase‐1 (PDK‐1) by AKT and the induction of hypoxia‐inducible factor‐1α (HIF‐1α). This process inhibits mitochondrial respiration and promotes glycolysis [53, 54]. BRAFi have been described to select for drug‐resistant, cycling, long‐term tumor‐maintaining melanoma cells. These cells express the H3K4‐demethylase JARID1B, responsible for higher levels of oxidative phosphorylation in melanoma‐resistant cells [55]. Our HRR data show that resistance to the BRAFi dabrafenib leads to mitochondrial dysfunction, including damage to the mitochondrial outer membrane, inhibition of glutamate metabolism, and dyscoupling of the electron transfer system with ATP production. TEM analysis of mitochondrial ultrastructure confirms these results and shows almost 50% of damaged mitochondria in the dabrafenib‐resistant cell line. This analysis also suggested that mitochondrial heterogeneity was more obvious between individual cells than within a given cell. The dysfunction of the damaged mitochondria is compensated by increased mitochondrial respiratory activity, resulting in higher mitochondrial membrane potential and ROS production in dabrafenib‐resistant cells. Interestingly, we observed that parental cells show reduced ROS scavenged levels by the antioxidant systems TrX and GSH. This reduction is not detected in dabrafenib‐resistant cells, suggesting that resistant cells could modulate TrX and GSH more efficiently, probably to overcome the toxic effects of higher ROS levels induced by the drug.

Commonly used approaches for the treatment of cancer, that is, radiation therapy, cytostatics, and also small molecule inhibitors of certain signaling molecules at least in part function through increasing ROS production. ROS as signaling molecules are implicated in normal cellular physiology through the process of redox modification [56]. However, excessive levels have been linked to damage to biomolecules and resulting cell death through various means [57]. It has been shown in the past that increasing ROS production through treatment with PEITC was more effective in killing oncogene‐transformed over non‐transformed cells [22]. Our results now show that this potency of PEITC is further enhanced in cells, that have acquired resistance to dabrafenib. Two additional compounds, which have been shown to increase mitochondrial ROS production, MitoPQ [42], and IACS [43], failed to show such an effect (MitoPQ) or failed to induce cell death (IACS) (data not shown). Antioxidant treatment substantially decreased PEITC‐induced cell killing strongly implying the requirement of ROS, but also indicating that probably other PEITC‐related effects may also be responsible for the observed effect [58]. In terms of clinical translation of these findings, PEITC may be of particular interest since it has already been shown that non‐toxic doses of PEITC (40 mg per day) are safe for human consumption and effective for therapeutic purposes [59].

We have previously established a link between p66Shc and ROS production in BRAFV600E transformed fibroblasts and melanoma cells [20]. PEITC‐induced increased ROS production was eliminated following pretreatment of cells with a JNK1/2 inhibitor. For BRAFV600E transformed fibroblasts, we demonstrated that shRNA‐mediated p66Shc knockdown decreased ROS production and enhanced proliferation in soft agar, which could be reproduced in A375 cells [20].

The data presented here further support a link between BRAF transformation and altered mitochondrial metabolism, which is involved in the development of BRAFi resistance, and which renders the affected melanoma cells sensitive to selective ROS‐inducing therapeutics.

## 5 Conclusion

In summary, we provide evidence for a specific mitochondrial phenotype of dabrafenib‐resistant melanoma cells, which results in constitutively increased ROS production. While this metabolic adaptation may enhance cellular viability through ROS effects on intracellular signaling, it also makes these cells more vulnerable for conditions, that further increase ROS levels. This may be exploited in future strategies for the treatment of BRAFi‐resistant melanoma.

## Conflict of interest

The authors declare no conflict of interest.

## Author contributions

SEa was responsible for conceptualization, data curation, formal analysis, investigation, methodology, project administration, supervision, validation, visualization, writing – original draft, and writing—review and editing. SEb was responsible for conceptualization, data curation, formal analysis, investigation, methodology, project administration, supervision, validation, visualization, and writing – review and editing. SEa and SEb are designated as equal contributors. CH conducted the investigation. JKG contributed to conceptualization, data curation, investigation, methodology, validation, and writing – review and editing. AD conducted the investigation. SS contributed to data curation, formal analysis, investigation, methodology, validation, and writing – review and editing. CV contributed to data curation, formal analysis, investigation, methodology, validation and writing – review and editing. AM contributed to data curation, formal analysis, investigation, and writing – review and editing. AN contributed to investigation, and methodology. AD contributed to investigation, and writing – review and editing. JES contributed to formal analysis, and investigation. MJB was responsible for data curation, formal analysis, investigation, resources, and writing – review and editing. MH: was responsible for data curation, investigation, resources, and writing – review and editing. JV conducted the investigation. GB contributed to review and editing and funding support. ES was involved in funding acquisition, investigation, project administration, resources, and writing – review and editing. OTQ contributed to conceptualization, data curation, formal analysis, funding acquisition, investigation, methodology, project administration, supervision, validation, and writing – review and editing. JT was involved in initial conceptualization, funding acquisition, methodology, project administration, resources, supervision, visualization, writing – original draft, and writing – review and editing.

## Consent

All authors have reviewed and approved the final version of this manuscript and consent to its submission and publication.

## Supporting information


**Fig. S1.** Effect of dabrafenib on the proliferation of A375P and A375D cells.
**Fig. S2.** A375D and WM164D cells show enhanced cellular respiration linked to mitochondrial dysfunction.
**Fig. S3.** Analysis of combined O_2_ and H_2_O_2_ fluxes on permeabilized A375P and A375D cells.
**Fig. S4.** IACS treatment is ineffective in inducing cell death and MitoPQ treatment does not increase mitochondrial ROS.
**Fig. S5.** Gating strategy for Annexin V/7AAD staining in A375P and A375D cells.
**Fig. S6.** Characterization of resistance in (A–C) WM164D and (D–F) 451LuD.
**Fig. S7.** Gating strategy for Annexin V/7AAD staining in WM164P and WM164D cells.
**Fig. S8.** Gating strategy for Annexin V/7AAD staining in 451LuP and 451LuD cells.
**Fig. S9.** Uncropped blots from the western blots for intracellular signaling in parental (A375P) and dabrafenib‐resistant (A375D) melanoma cells.

## Data Availability

The datasets generated during and/or analyzed during the current study are available from the corresponding author on reasonable request.
