# Supplementary material for: Exploiting metabolic adaptations to overcome dabrafenib treatment resistance in melanoma cells
Source: Mol Oncol. 2025 Dec 2;20(5):1202–19. doi: 10.1002/1878-0261.70169 (PMC13155145; doi:10.1002/1878-0261.70169)
Supplement: Supplementary file 1 — Fig. S1. Effect of dabrafenib on the proliferation of A375P and A375D cells. Fig. S2. A375D and WM164D cells show enhanced cellular respiration linked to mitochondrial dysfunction. Fig. S3. Analysis of combined O2 and H2O2 fluxes on permeabilized A375P and A375D cells. Fig. S4. IACS treatment is ineffective in inducing cell death and MitoPQ treatment does not increase mitochondrial ROS. Fig. S5. Gating strategy for Annexin V/7AAD staining in A375P and A375D cells. Fig. S6. Characterization of resistance in (A–C) WM164D and (D–F) 451LuD. Fig. S7. Gating strategy for Annexin V/7AAD staining in WM164P and WM164D cells. Fig. S8. Gating strategy for Annexin V/7AAD staining in 451LuP and 451LuD cells. Fig. S9. Uncropped blots from the western blots for intracellular signaling in parental (A375P) and dabrafenib‐resistant (A375D) melanoma cells. [file MOL2-20-1202-s001.pdf]

SUPPLEMENTARY INFORMATION

Exploiting metabolic adaptations to overcome dabrafenib treatment resistance in melanoma cells

Silvia Eller[1*], Susanne Ebner[1*], Carmen Haselrieder[1], Julia K Günther[1], Astrid Drasche[1], Sophie Strich[2,3], Chiara Volani[4], Andrea Medici[1], Aleksandar Nikolajevic[1], Alex Deltedesco[1], Johannes E Sigmund[1], Michael J Blumer[5], Martin Hermann[6], Johanna Vanacker[1], Gerald Brandacher[1], Eduard Stefan[2,3], Omar Torres-Quesada[7,3], Jakob Troppmair[1]

[1]Daniel Swarovski Research Laboratory, Department of Visceral, Transplant and Thoracic Surgery, Medical University of Innsbruck, Austria, [2]Institute of Molecular Biology and Center for Molecular Biosciences Innsbruck (CMBI), University of Innsbruck, Austria, [3]Tyrolean Cancer Research Institute (TKFI), Innsbruck, Austria, [4]University Clinic for Internal Medicine II, Medical University of Innsbruck, Austria, [5]Department of Anatomy, Histology and Embryology, Institute of Clinical and Functional Anatomy, Medical University of Innsbruck, Austria, [6]Department of Anaesthesia and Intensive Care Medicine, Medical University of Innsbruck, Austria, [7]Division of Medical Biochemistry, Biocenter, Medical University of Innsbruck, Austria

* These authors contributed equally to this work.

Corresponding authors:

Jakob Troppmair, PhD
Daniel Swarovski Research Laboratory
Department of Visceral, Transplant and Thoracic Surgery
Medical University Innsbruck
Innrain 66
6020 Innsbruck, Austria
E-Mail: jakob.troppmair@i-med.ac.at

Omar Torres-Quesada, PhD
Division of Medical Biochemistry
Biocenter
Medical University of Innsbruck
Innrain 80/82
6020 Innsbruck, Austria
E-Mail: omar.torres-quesada@i-med.ac.at
and
Tyrolean Cancer Research Institute (TKFI)
Innrain 66
6020 Innsbruck, Austria

This file contains:

Supplementary figure 1 till 9

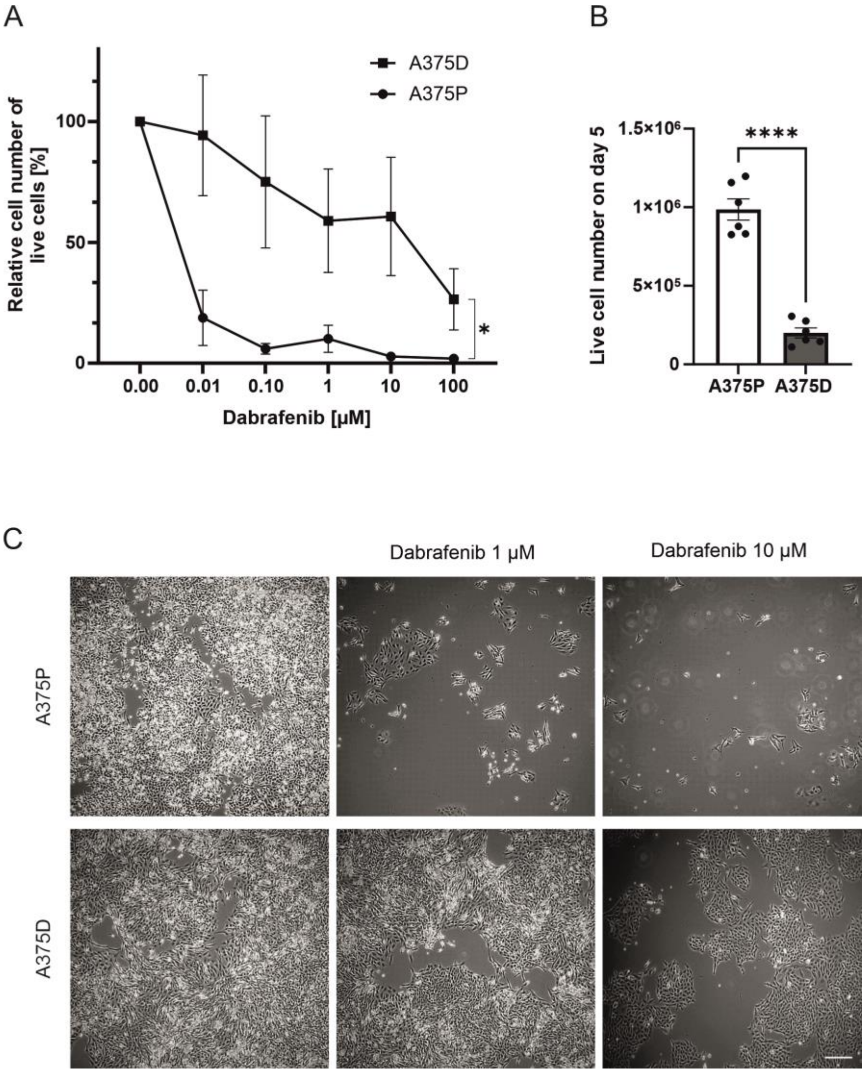

Supplementary Figure 1: Effect of dabrafenib on the proliferation of A375P and A375D cells. To assess cell viability A375P and A375D cells were seeded in complete medium at a density of 3 x 10^3 – 5 x 10^3 cells per well in 6-well plates. The next day the cells were exposed to increasing concentrations of dabrafenib: 0 µM, 1.5 µM, 10 µM. After a 5-day cultivation period, the medium from each well was collected to ensure that any floating cells were not lost. The remaining adherent cells were then washed with PBS and detached by trypsinization using TrypLE Express (Gibco) and centrifuged at 425 x g for 10 minutes. Subsequently, the number of viable cells was determined via trypan blue exclusion assay. For this, the cells were stained with Trypan Blue (T8154, Sigma-Aldrich) and counted using a Neubauer-improved counting chamber (Marienfeld Superior). Microscopic images were also captured after 5 days. (A) A375P and A375D cells were exposed to varying concentrations of dabrafenib (0.01, 0.1, 1, 10 and 100 µM) for five days, alongside a DMSO control group. Data were normalized to the respective DMSO control group of A375P and A375D cells and are presented as percent

of mean ± SEM; Area Under the Curve (AUC) test was performed to analyze the dose-response of dabrafenib on cell proliferation, and statistical significance was assessed using an unpaired t-test; N = 5. (*$p < 0.05$) (B) Live cell numbers of untreated A375P and A375D cells after 5 days are shown. Initially, cells were seeded in 6-well plates with a density of 5,000 cells/well. This resulted in average live cell numbers of 986,042 (SD 151,263) for untreated A375P and 200,083 (SD 70,528) for untreated A375D cells, indicating a significantly slower proliferative rate of A375D cells as a characteristic compared to A375P. Statistical significance was determined using an unpaired t-test; N = 6. (**** $p \leq 0.0001$) (C) Microscopic images captured after five days of dabrafenib treatment (1 µM and 10 µM) visualize the effect on cell proliferation of A375P and A375D cells; N=2. Scale bar = 200 µm.

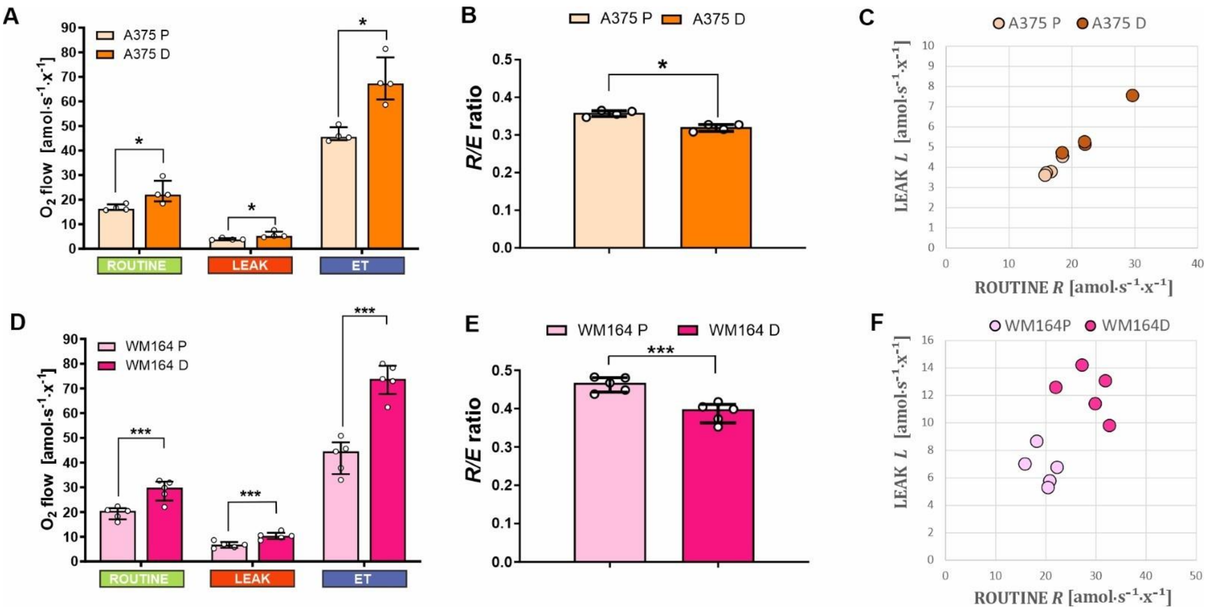

Supplementary Figure 2: A375D and WM164D cells show enhanced cellular respiration linked to mitochondrial dysfunction. High resolution respirometry (HRR) experiments of A375 and WM164 cells using the SUIT-003-O2 ce D009 protocol, which has been designed for studying the respiratory capacities ROUTINE, LEAK and ET in non-permeabilized cells (living cells). **A.** O_2 consumption analysis by HRR in A375P and A375D representing the respiratory coupling control states [amol·s^{-1}·x^{-1}] baseline-corrected for residual O_2 consumption Rox. "x" represents the unit cell. **B.** *R/E* respiratory control ratio representing the ROUTINE *R* respiration normalized to maximum ET capacity *E*. **C.** Bioenergetic cluster analysis of the ROUTINE and LEAK respiratory coupling states showing the two bioenergetic clusters where the A375D cells show higher dyscoupled respiration LEAK (Gnaiger 2021). **D.** O_2 consumption analysis by HRR in WM164P and WM164D representing the respiratory coupling control states [amol·s^{-1}·x^{-1}] baseline-corrected for residual O_2 consumption Rox. "x" represents the unit cell. **E.** *R/E* respiratory control ratio representing the ROUTINE *R* respiration normalized to maximum ET capacity *E*. **F.** Bioenergetic cluster analysis of the ROUTINE and LEAK respiratory coupling states showing the two bioenergetic clusters where the WM164D cells show higher dyscoupled respiration LEAK (Gnaiger, 2021, https://doi.org/10.26124/mitofit:2021-0008). Results are represented as median ± IQR (50 % range). 2-way ANOVA with Bonferroni multiple comparison test or non-parametric unpaired t-test analysis were performed to assess significance; N= 6 (* $p \leq 0.05$; *** $p \leq 0.001$). ET, electron-transfer-pathway capacity.

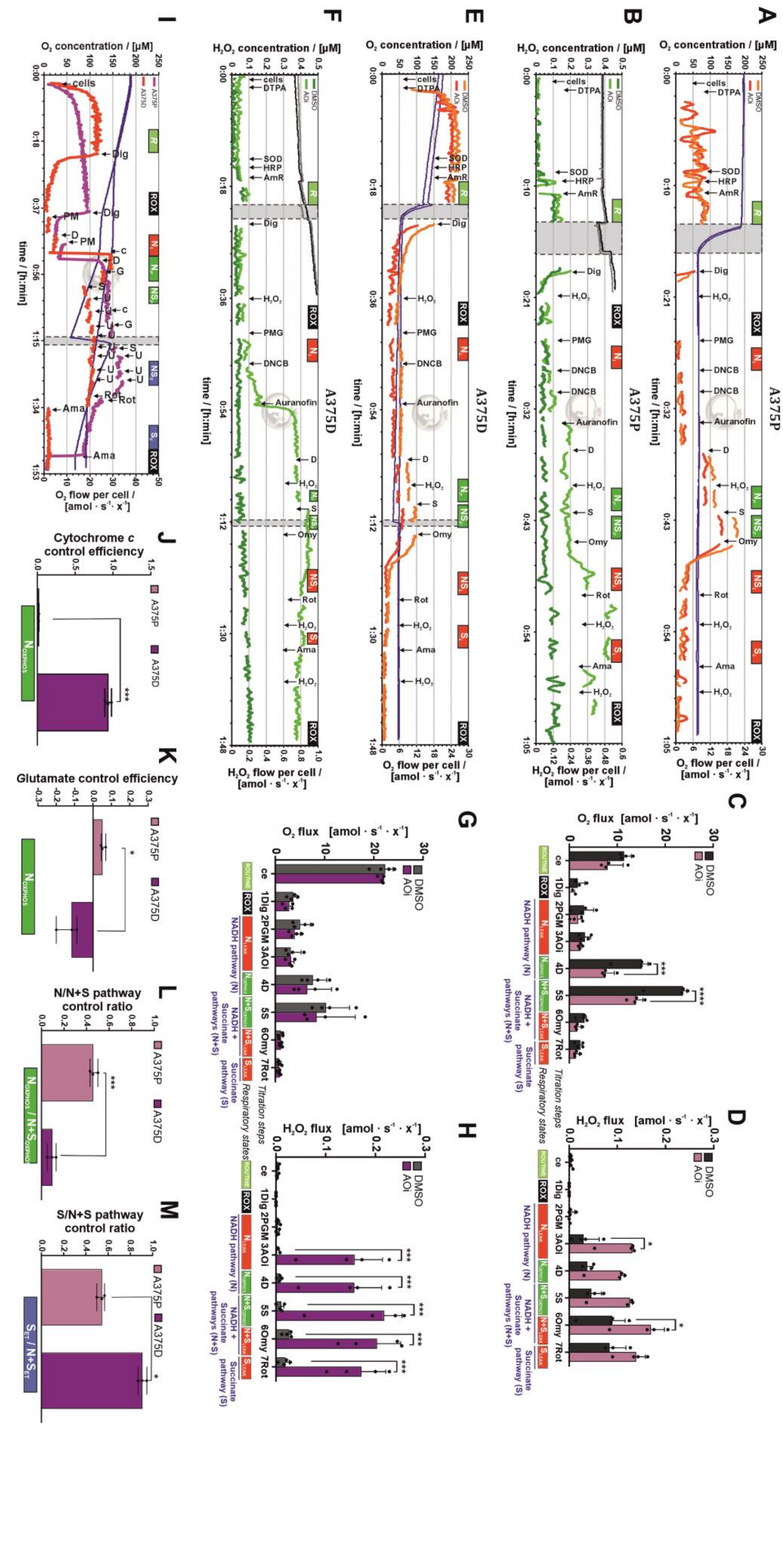
A
A375P
B
A375P
C
D
E
A375D
F
A375D
G
H
I
J
Cytochrome c control efficiency
K
Glutamate control efficiency
L
N/N+S pathway control ratio
M
S/N+S pathway control ratio

Supplementary Figure 3: Analysis of combined O_2 and H_2O_2 fluxes on permeabilized A375P and A375D cells. Representative respiratory traces for the protocol SUIT-DLPu O2 pce AmR of simultaneous measurements of O_2 and H_2O_2 fluxes of A375P (A, B) and A375D (E, F) cells. Blue lines: O_2 concentration [μM]; red line: O_2 flow per cell [amol•s^{-1}•x^{-1}]. "x" represents the unit cell. (C, G) Analysis of the O_2 fluxes and (D, H) H_2O_2 fluxes are shown for each experimental group. Sequential steps: *R*; Routine; Dig: digitonin; PMG: pyruvate, malate, glutamate; AOi: antioxidant system inhibitors Auranofin and DNCB; D: ADP; S: succinate; Omy: oligomycin; Rot: rotenone; Ama: antinomycin A. (I) Representative respiratory traces for the protocol SUIT-008 O2 pce D025 in A375 parental and dabrafenib resistant cells. Blue lines: O_2 concentration [μM]; red line: O2 flow per cell amol•s^{-1}•x^{-1}] in A375P and pink line: O_2 flow per cell [amol•s^{-1}•x^{-1} in A375D. "x" represents the unit cell. Sequential steps: Dig: digitonin; PM: pyruvate, malate; D: ADP; c: cytochrome *c*; G: glutamate; S: succinate; U: CCCP; Rot: rotenone; U: uncoupler CCCP; Ama: antimycin A. (J) Cytochrome c control efficiency = (2*c*-2*D*)/2*c* (K) Glutamate control efficiency = (3*G*-2*c*)/3*G*. (L) N/NS pathway control ratio. (M) S/NS pathway control ratio. *R*, ROUTINE respiration; *L*: LEAK; *P*: OXPHOS; *E*: ET capacity; ROX: residual oxygen consumption; N: NADH electron transfer-pathway state; S: Succinate pathway; NS: NS-pathway. Results are represented as median ± IQR (50 % range). 2-way ANOVA with Bonferroni multiple comparison test or non-parametric unpaired t-test analysis; N = 4. (* $p \leq 0.05$; ** $p \leq 0.01$; *** $p \leq 0.001$).

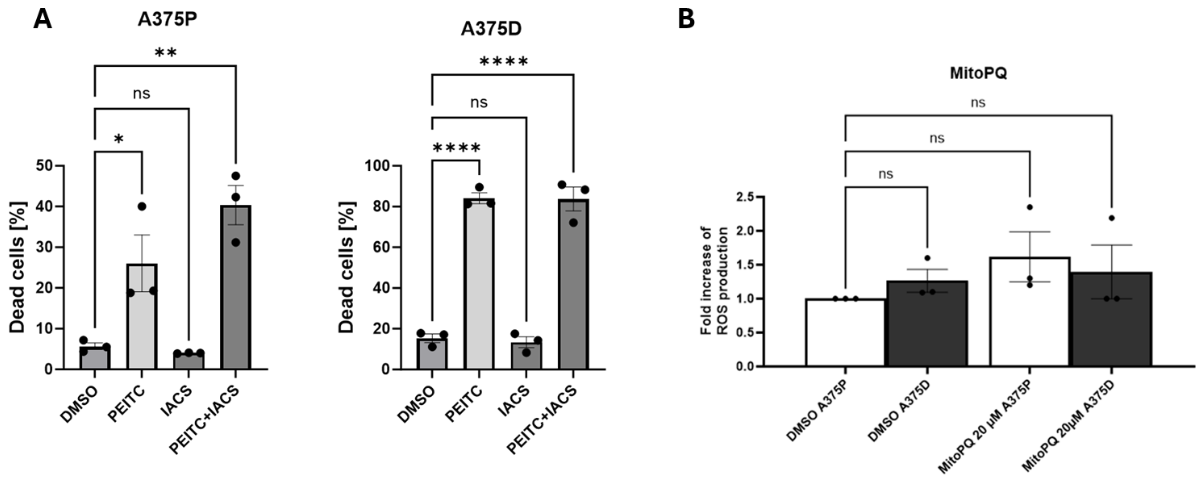

Supplementary Figure 4: IACS treatment is ineffective in inducing cell death and MitoPQ treatment does not increase mitochondrial ROS. (A) A375P and A375D cells were seeded in six-well plates at a density of 1 × 10^5 cells per well. 24 hours later, cells were treated with 7.5 µM PEITC and/or 1 µM IACS for 16 hours. Cell death was assessed by flow cytometry using Annexin V/7AAD staining. The percentage of dead cells is displayed. Data are presented as mean ± SEM. Statistically significant differences between groups were tested using ordinary one-way ANOVA; N=3. (* $p \leq 0.05$; ** $p \leq 0.01$; **** $p \leq 0.0001$). (B) 2 x 10^5 A375P and A375D cells were seeded per well in 8-well Nunc Lab-Tek chambers. After 24 hours, cells were treated with 20 µM MitoPQ for 30 minutes and subsequently stained with MitoTracker Red CM-H2XRos to assess mitochondrial ROS levels. Quantification of ROS levels is shown. Data are presented as mean ± SEM. Statistically significant differences between groups were tested using ordinary one-way ANOVA; N = 3. (ns, not significant).

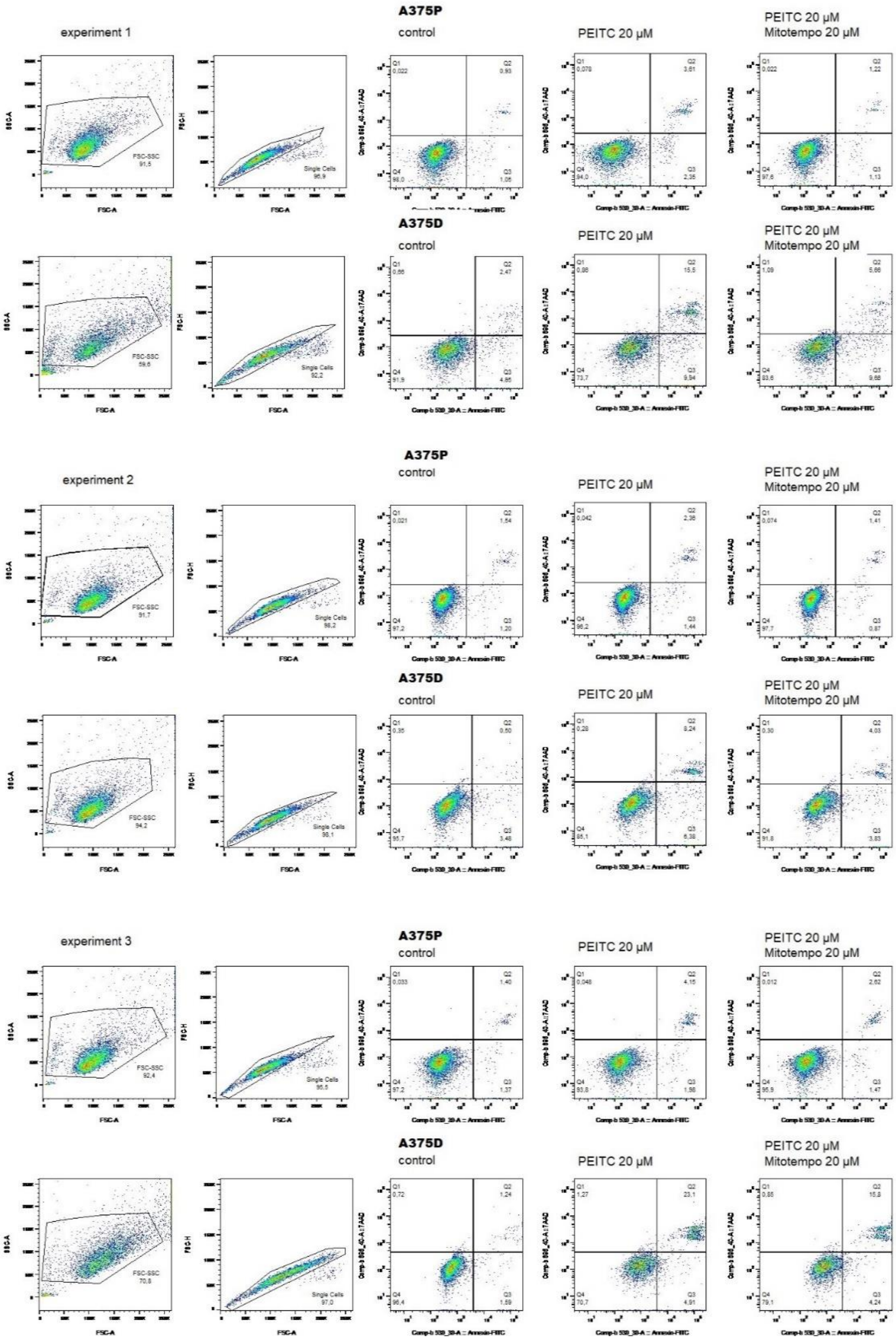
experiment 1
A375P
control
PEITC 20 µM
PEITC 20 µM
Mitotempo 20 µM
A375D
control
PEITC 20 µM
PEITC 20 µM
Mitotempo 20 µM
experiment 2
A375P
control
PEITC 20 µM
PEITC 20 µM
Mitotempo 20 µM
A375D
control
PEITC 20 µM
PEITC 20 µM
Mitotempo 20 µM
experiment 3
A375P
control
PEITC 20 µM
PEITC 20 µM
Mitotempo 20 µM
A375D
control
PEITC 20 µM
PEITC 20 µM
Mitotempo 20 µM
FSC-A
SSC-A
FSC-H
FSC-SSC
Single Cells

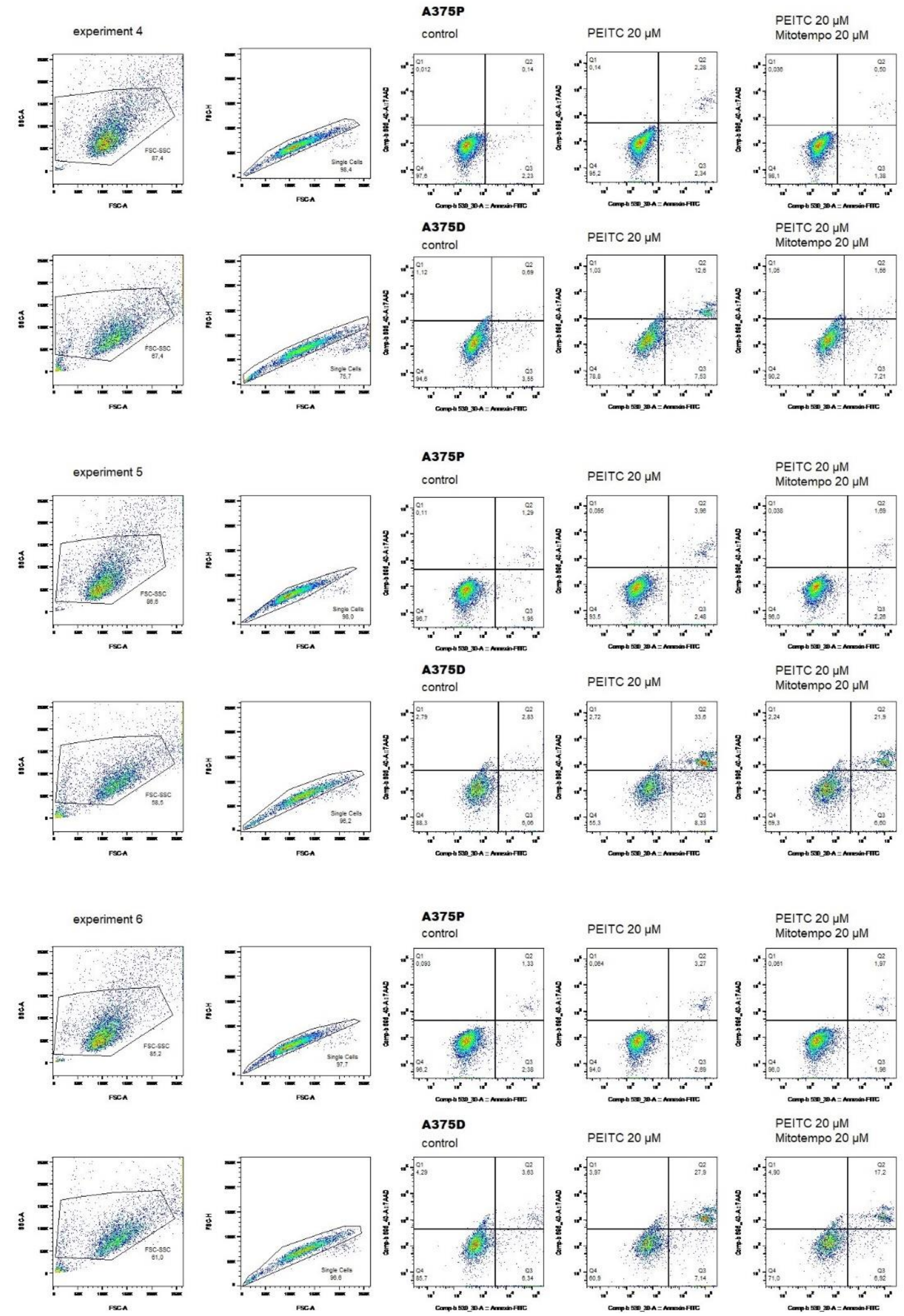

Supplementary Figure 5: Gating strategy for Annexin V/7AAD staining in A375P and A375D cells. A375 cells were first gated using **an** FSC-A vs. SSC-A dot plot to isolate the main cell population, followed by doublet exclusion via FSC-A vs. FSC-H dot plots. Identical gating was applied for both A375P and A375D cells. The final dot plots display Annexin V-FITC (x-axis) vs. 7AAD (y-axis) fluorescence. Cells were treated with 20 µM PEITC for 30 minutes alone or pretreated with 20 µM MitoTempo for 16 hours followed by 30 minutes of PEITC stimulation. N=6.

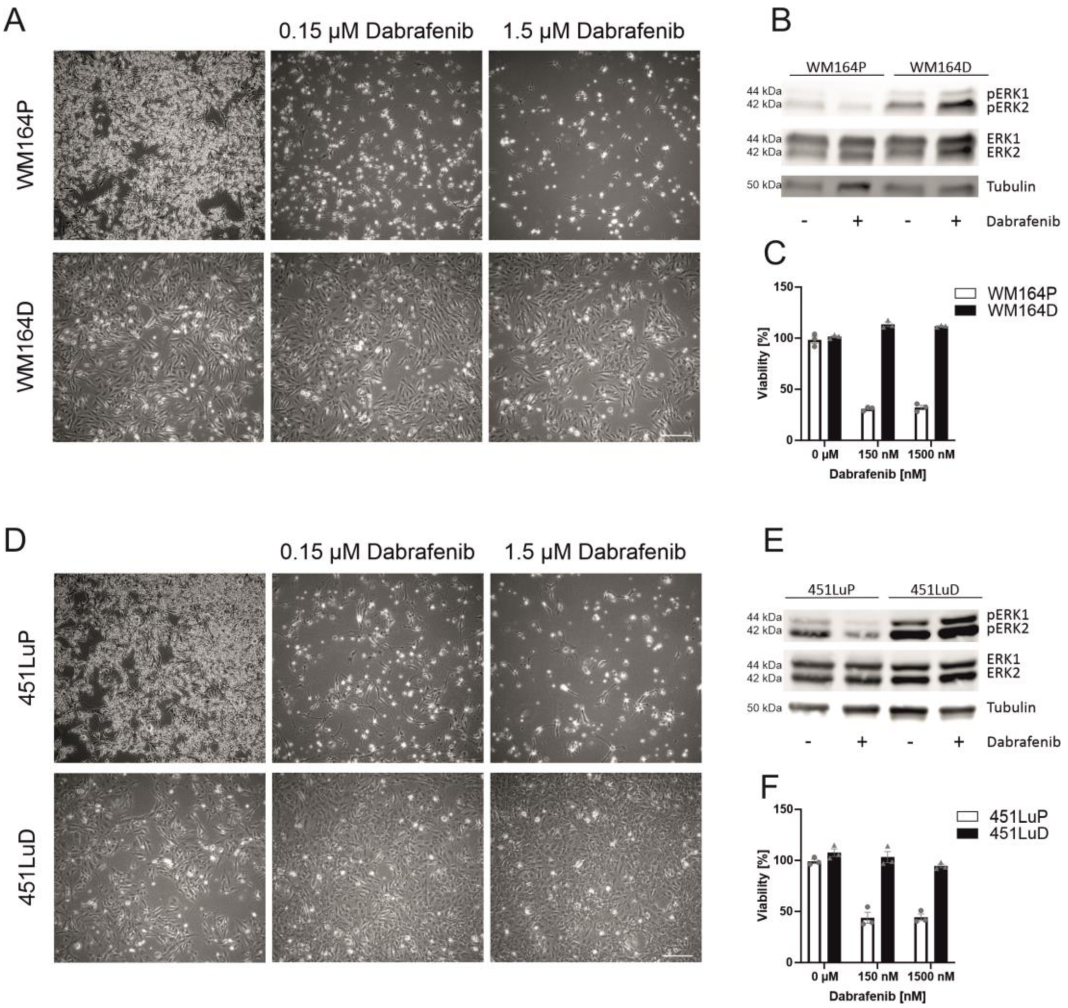

Supplementary Figure 6: Characterization of resistance in (A-C) WM164D and (D-F) 451LuD

Parental and dabrafenib-resistant WM164 and 451Lu cells were seeded at a density of 5 x 10^3 per well in 96-well plates and allowed to grow for 48 hours before growth was assessed via the WST-8 assay (ab228554, abcam) as previously described [25]. For microspcopic imaging, WM164 and 451Lu cells were seeded at a density of 2 x 10^5 per well in 6-well plates. After 24 hours, WM164 and 451Lu cells were treated with DMSO, 150 nM and 1.5 µM dabrafenib and allowed to grow for 72 hours prior to imaging. (A) Microscopic images of WM164P and WM164D cells after 72 hours of treatment with dabrafenib (0.15 µM and 1.5 µM). (B) Immunoblots conducted with antibodies specific for phosphorylated and total ERK after 3 hours of dabrafenib treatment. (C) Cell viability assessed with WST-8 assay after 48 hours of dabrafenib treatment (0.15 µM and 1.5 µM); N=2-3. (D) Microscopic images of 451LuP and 451LuD cells after 72 hours of treatment with dabrafenib (0.15 µM and 1.5 µM). (E) Immunoblots carried out with antibodies specific for phosphorylated and total ERK after 3

hours of dabrafenib treatment. (F) Cell viability assessed with WST-8 assay after 48 hours of dabrafenib treatment (0.15 µM and 1.5 µM); N=3. Scale bar = 200 µm.

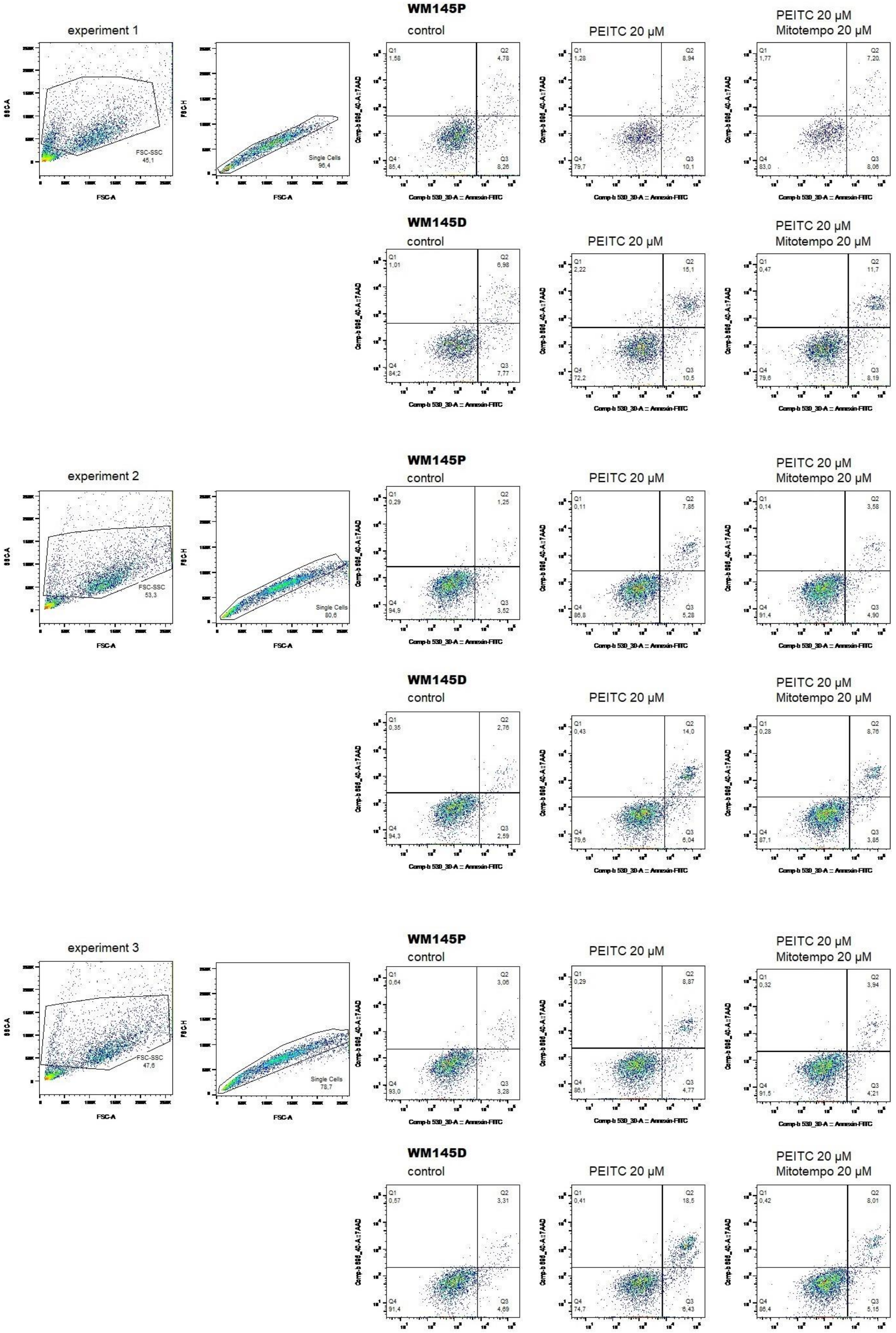

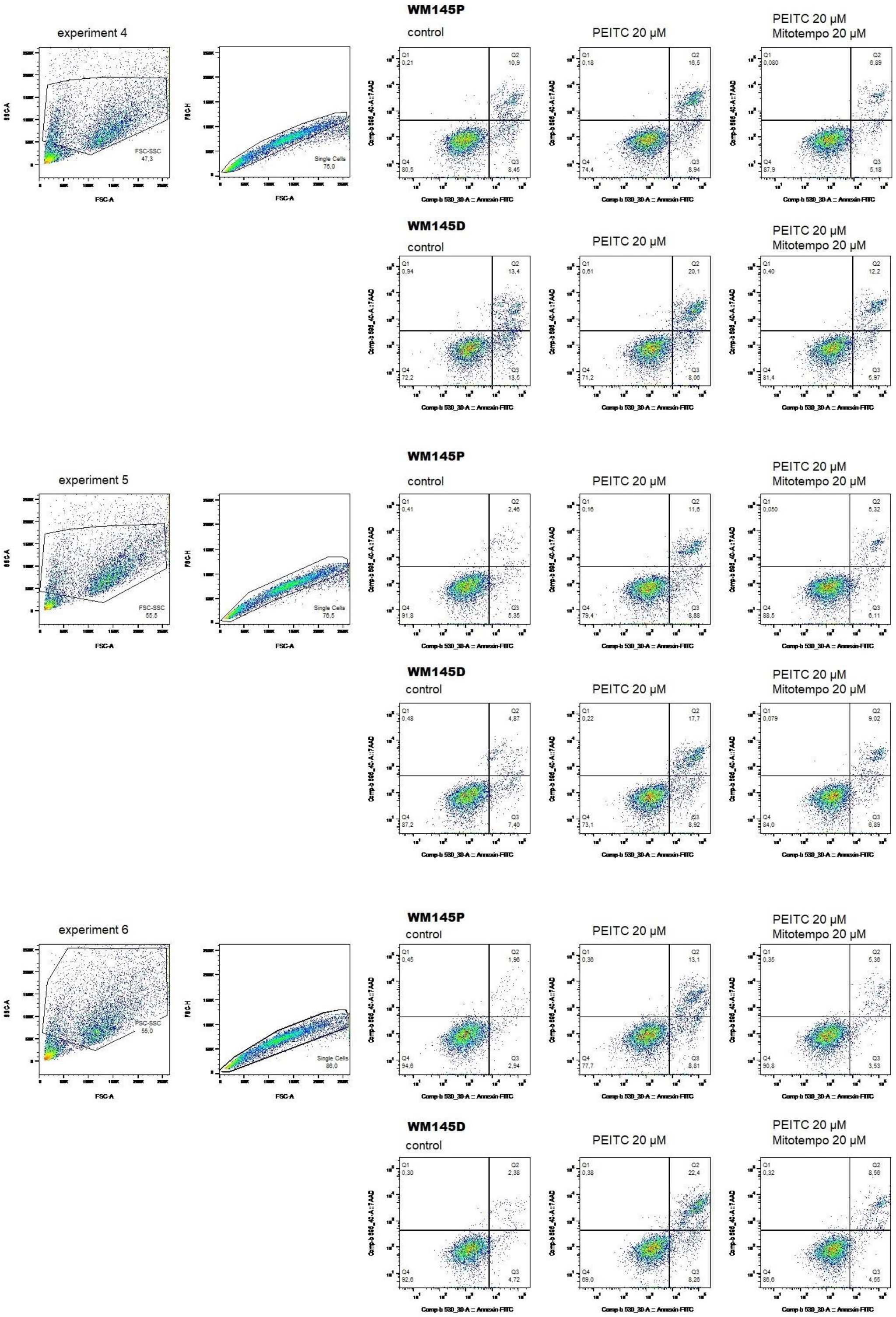
experiment 4
WM145P
control
PEITC 20 µM
PEITC 20 µM
Mitotempo 20 µM
WM145D
control
PEITC 20 µM
PEITC 20 µM
Mitotempo 20 µM
experiment 5
WM145P
control
PEITC 20 µM
PEITC 20 µM
Mitotempo 20 µM
WM145D
control
PEITC 20 µM
PEITC 20 µM
Mitotempo 20 µM
experiment 6
WM145P
control
PEITC 20 µM
PEITC 20 µM
Mitotempo 20 µM
WM145D
control
PEITC 20 µM
PEITC 20 µM
Mitotempo 20 µM

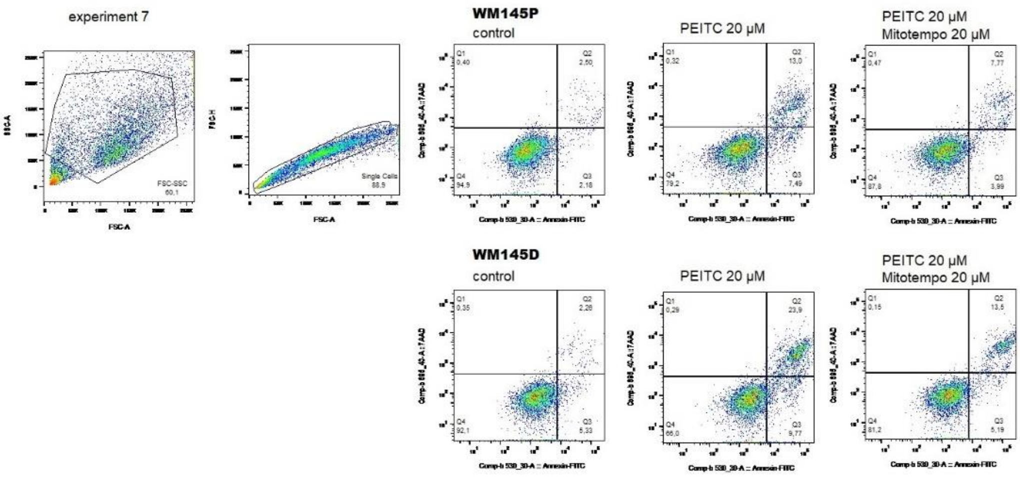

Supplementary Figure 7: Gating strategy for Annexin V/7AAD staining in WM164P and WM164D cells. WM164 cells were first gated using an FSC-A vs. SSC-A dot plot to isolate the main cell population, followed by doublet exclusion via FSC-A vs. FSC-H dot plots. Identical gating was applied for both WM164P and WM164D cells. The final dot plots display Annexin V-FITC (x-axis) vs. 7AAD (y-axis) fluorescence. Cells were treated with 20 µM PEITC for 1.5 hours alone or pretreated with 20 µM MitoTempo for 16 hours followed by 1.5 hours of PEITC stimulation. N=7.

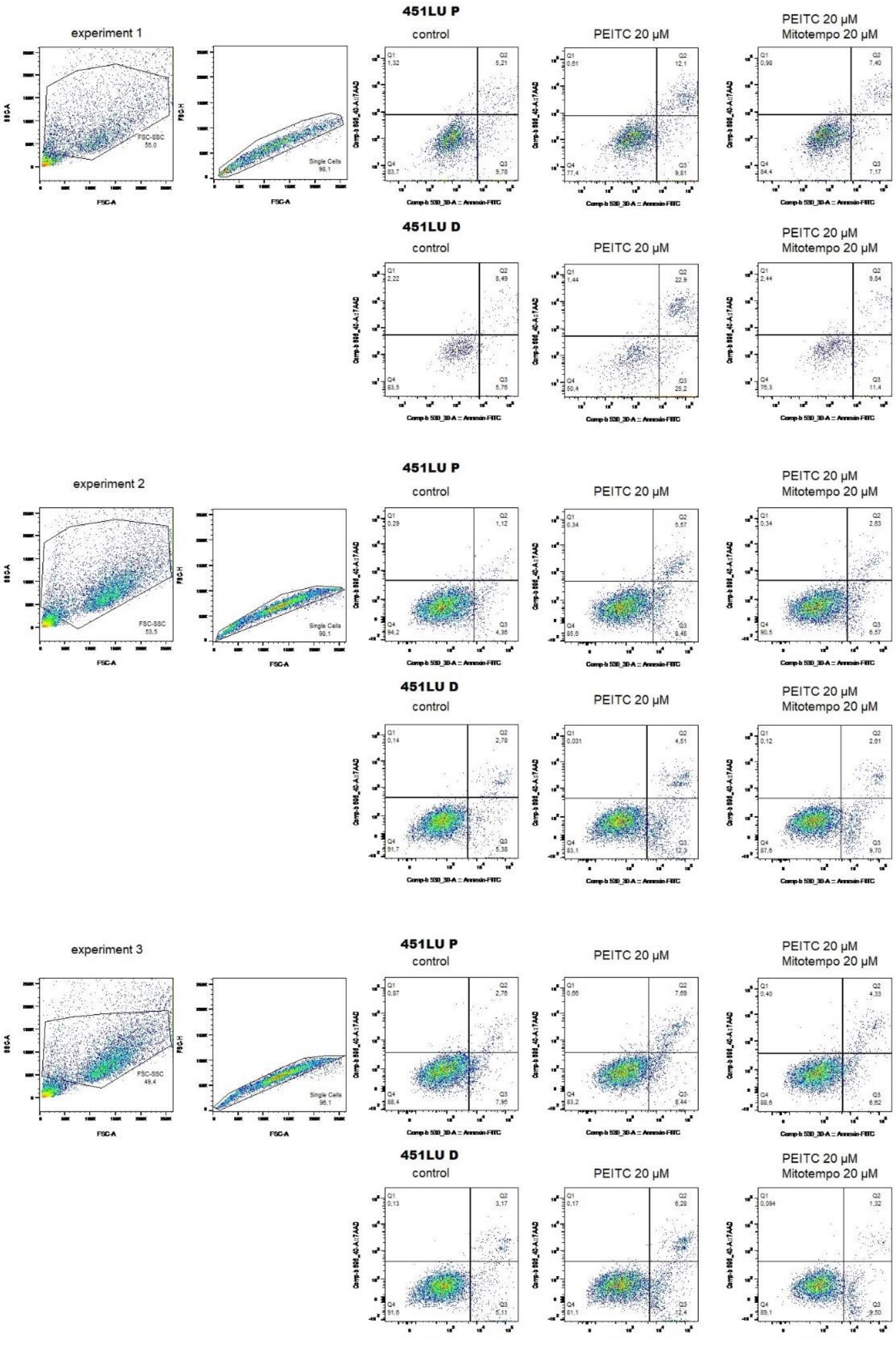
experiment 1
experiment 2
experiment 3
451LU P
451LU D
control
PEITC 20 µM
PEITC 20 µM
Mitotempo 20 µM
FSC-A
SSC-A
FSC-H
Single Cells
FSC-SSC
Comp-b 530_30-A :: Annexin-FITC

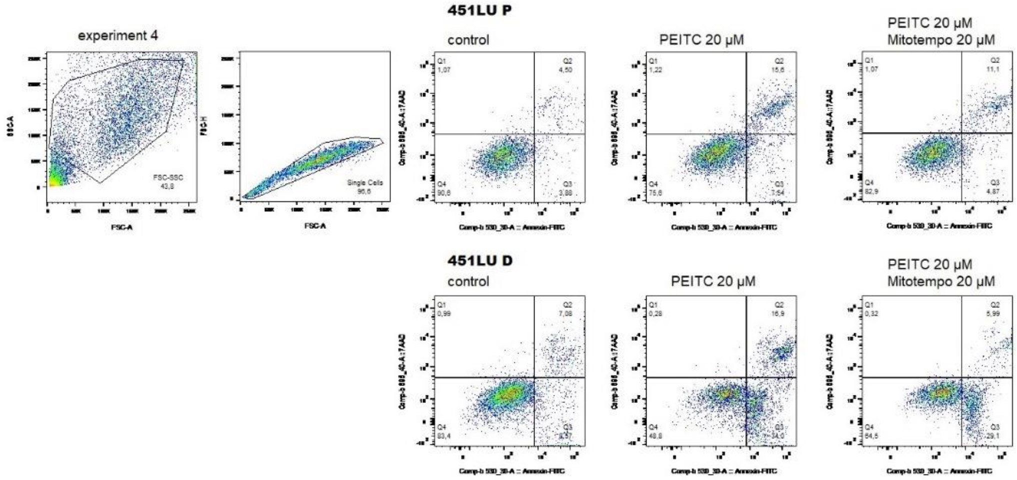
experiment 4
451LU P
control
PEITC 20 µM
PEITC 20 µM
Mitotempo 20 µM
451LU D
control
PEITC 20 µM
PEITC 20 µM
Mitotempo 20 µM

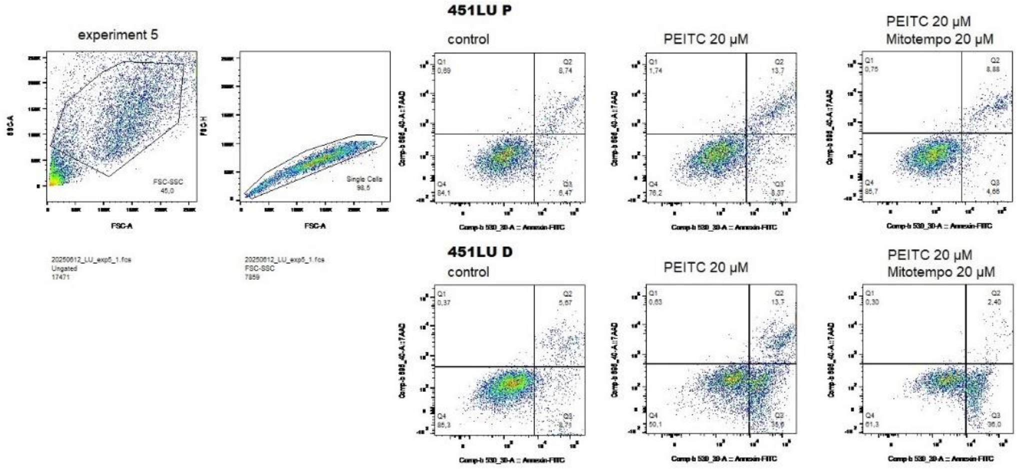
experiment 5
451LU P
control
PEITC 20 µM
PEITC 20 µM
Mitotempo 20 µM
451LU D
control
PEITC 20 µM
PEITC 20 µM
Mitotempo 20 µM

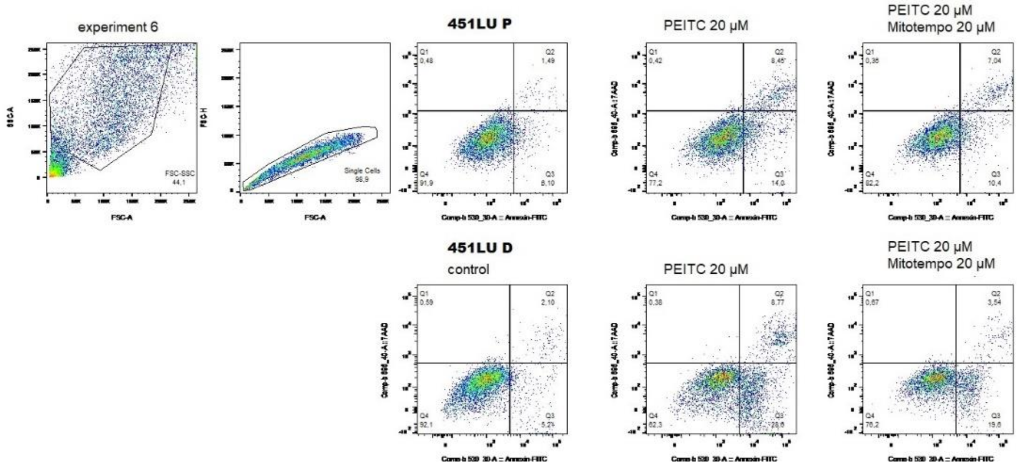
experiment 6
451LU P
PEITC 20 µM
PEITC 20 µM
Mitotempo 20 µM
451LU D
control
PEITC 20 µM
PEITC 20 µM
Mitotempo 20 µM

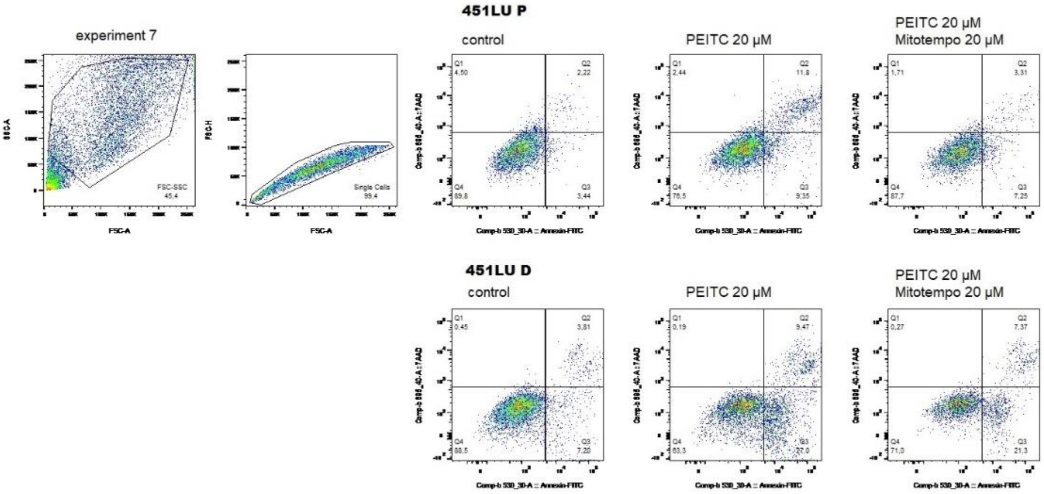

Supplementary Figure 8: Gating strategy for Annexin V/7AAD staining in 451LuP and 451LuD cells. 451Lu cells were first gated using an FSC-A vs. SSC-A dot plot to isolate the main cell population, followed by doublet exclusion via FSC-A vs. FSC-H dot plots. Identical gating was applied for both 451LuP and 451LuD cells. The final dot plots display Annexin V-FITC (x-axis) vs. 7AAD (y-axis) fluorescence. Cells were treated with 20 µM PEITC for 2 hours alone or pretreated with 20 µM MitoTempo for 16 hours followed by 2 hours of PEITC stimulation. N=7.

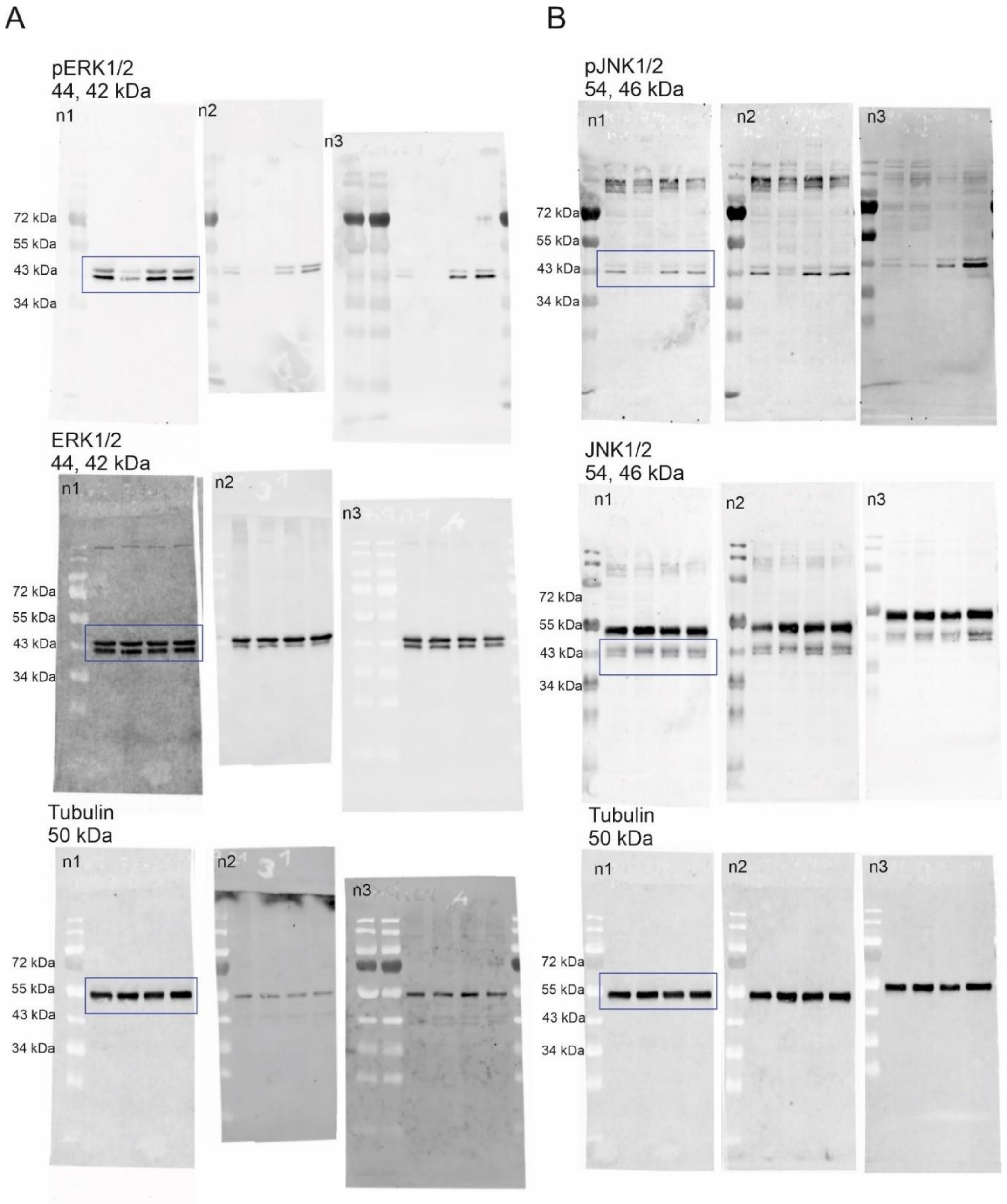
A
pERK1/2
44, 42 kDa
n1
n2
n3
72 kDa
55 kDa
43 kDa
34 kDa
ERK1/2
44, 42 kDa
n1
n2
n3
72 kDa
55 kDa
43 kDa
34 kDa
Tubulin
50 kDa
n1
n2
n3
72 kDa
55 kDa
43 kDa
34 kDa
B
pJNK1/2
54, 46 kDa
n1
n2
n3
72 kDa
55 kDa
43 kDa
34 kDa
JNK1/2
54, 46 kDa
n1
n2
n3
72 kDa
55 kDa
43 kDa
34 kDa
Tubulin
50 kDa
n1
n2
n3
72 kDa
55 kDa
43 kDa
34 kDa

C

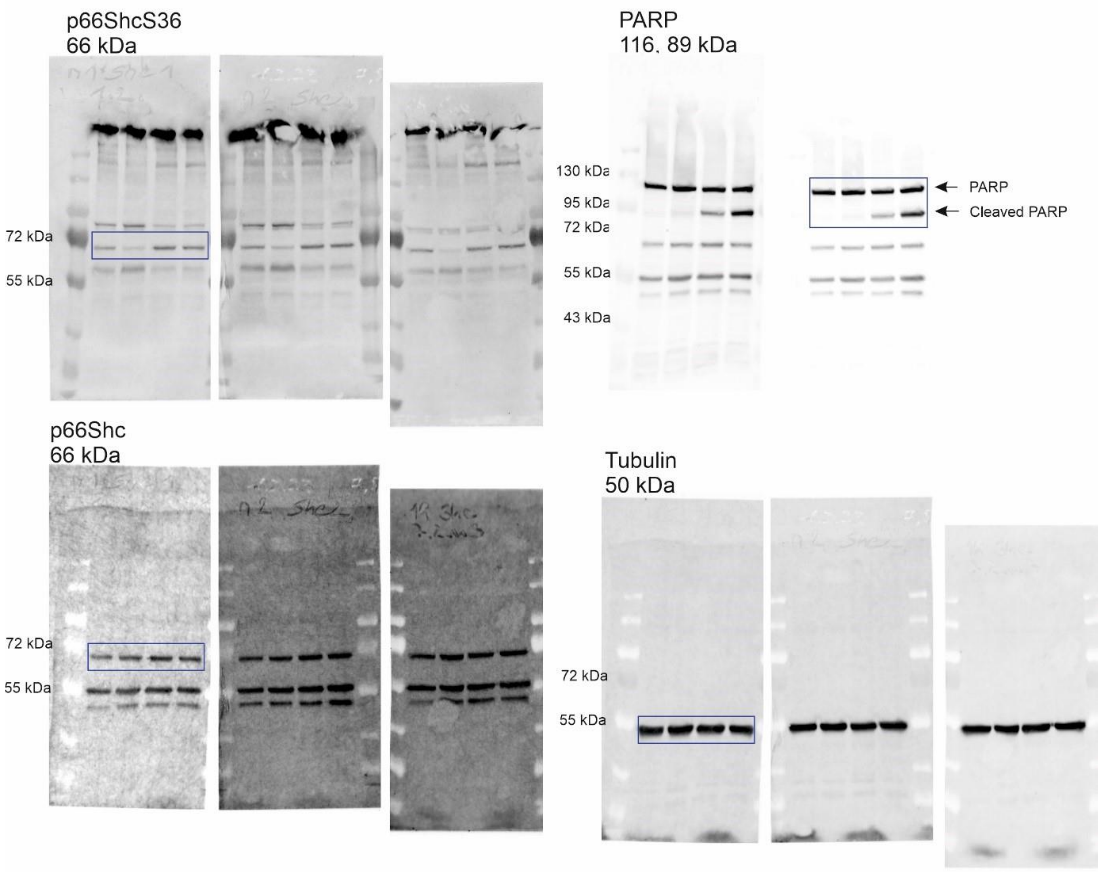
p66ShcS36
66 kDa
72 kDa
55 kDa
PARP
116, 89 kDa
130 kDa
95 kDa
72 kDa
55 kDa
43 kDa
PARP
Cleaved PARP
p66Shc
66 kDa
72 kDa
55 kDa
Tubulin
50 kDa
72 kDa
55 kDa

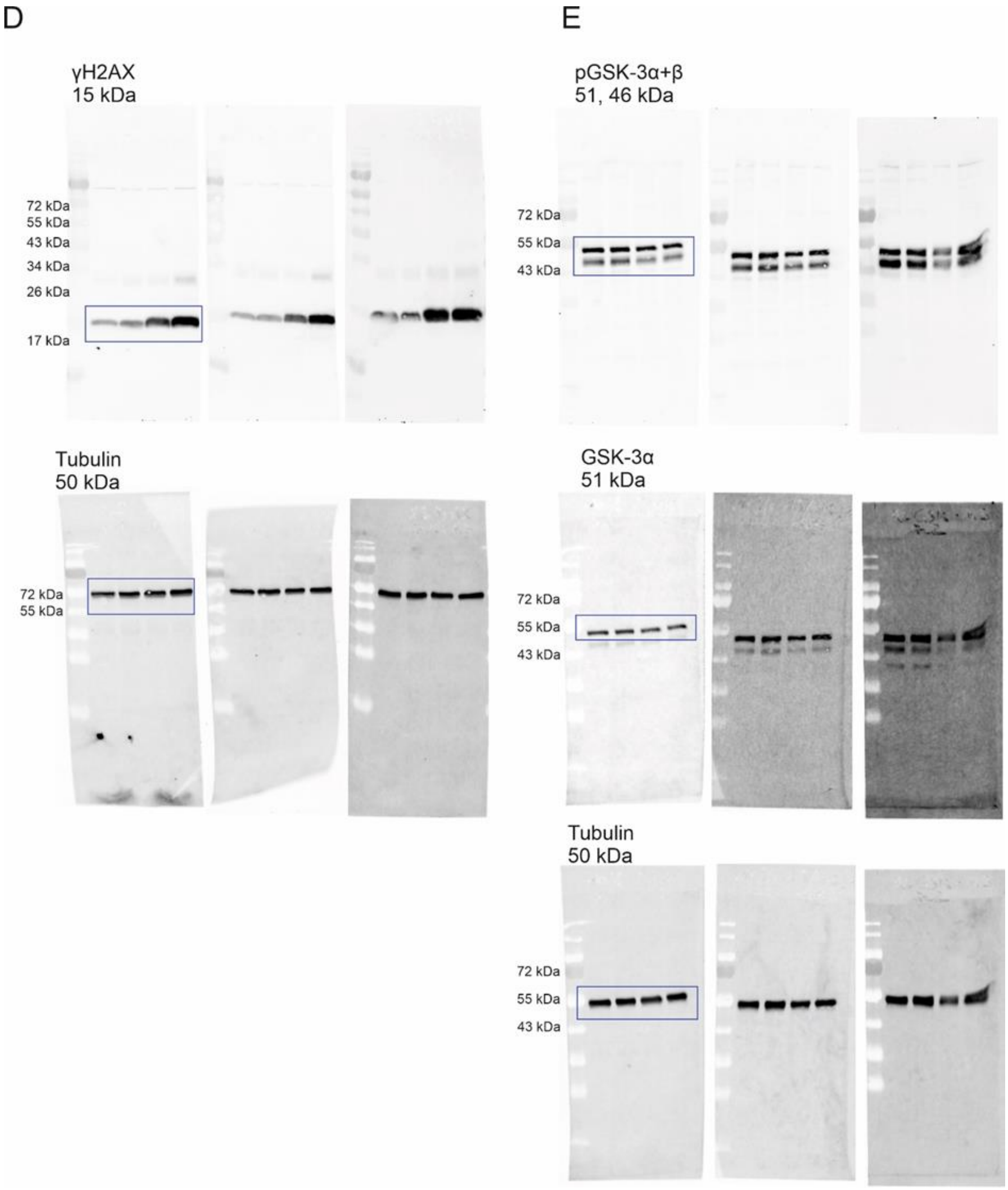

Supplementary Figure 9: Uncropped blots from the western blots for intracellular signaling in parental (A375P) and dabrafenib-resistant (A375D) melanoma cells. This figure corresponds to the blots presented in Figure 1 of the main text and provides a complete representation of protein expression levels from 2–3 independent experiments. The specific lanes used in the main manuscript are highlighted in blue for easy reference. The lane assignments are as follows: Lane 1: A375P treated with 1.5 µM DMSO, Lane 2: A375P treated with 1.5 µM Dabrafenib, Lane 3: A375D treated with 1.5 µM DMSO, Lane 4: A375D treated with 1.5 µM Dabrafenib. Immunoblots were conducted using antibodies specific for (A) pERK1/2, ERK1/2, and Tubulin; (B) pJNK1/2, JNK1/2, and Tubulin; (C) p66ShcS36, p66Shc, PARP, and Tubulin; (D) γH2AX and Tubulin; and (E) pGSK-3α/β, GSK-3α, and Tubulin. For clarification, in immunoblot (C), all the named antibodies (p66ShcS36, p66Shc, PARP, and Tubulin) were probed sequentially on the same blot.
